# Tetrazine-based bio-protection: reversible masking of cell surface thiols for orthogonal cellular chemistry

**DOI:** 10.1039/d6sc04306b

**Published:** 2026-11-03

**Authors:** Mingchen Sun, Katerina Gavriel, Chenxuan Wan, Jelte Schermer, Kevin Neumann, Daniela A. Wilson

**Affiliations:** a Radboud University Nijmegen, Institute for Molecules and Materials Heyendaalseweg 135 6525 AJ Nijmegen The Netherlands d.wilson@science.ru.nl kevin.neumann@ru.nl

## Abstract

Cell-surface thiols are redox-sensitive functional groups that regulate cellular signalling, surface reactivity, and thiol-mediated uptake. However, these thiols are highly susceptible to irreversible oxidation during oxidative surface modification procedures, compromising cell viability and functionalization precision. Here, we report a reversible and biocompatible thiol protection strategy using sulfide tetrazines that covalently mask exofacial thiols on live cells *via* rapid nucleophilic aromatic substitution. This masking renders the thiols inert to oxidative damage, as demonstrated in both polymeric membranes and viable Jurkat and HeLa cells, where tetrazine-treated surfaces resisted NaIO_4_^−^ induced thiol oxidation. Importantly, the protection is readily reversed by extracellular treatment with glutathione, thus restoring native thiols *via* nucleophilic aromatic substitution. Furthermore, we show that tetrazine-based protection preserves thiol-mediated cellular uptake capabilities, enabling precise temporal control over cell-surface thiol reactivity. This bio-protection strategy extends the toolkit of bioorthogonal chemistry to conditional masking of thiols, opening new avenues for dynamic cell-surface engineering, redox biology investigations, and controlled bioconjugation strategies in complex cellular environments.

## Abstract 2

Tetrazine-based protection transiently masks cell-surface thiols, shielding them from oxidative damage. GSH-mediated deprotection restores native thiol reactivity and preserves thiol-mediated cellular uptake.

## Graphical abstract

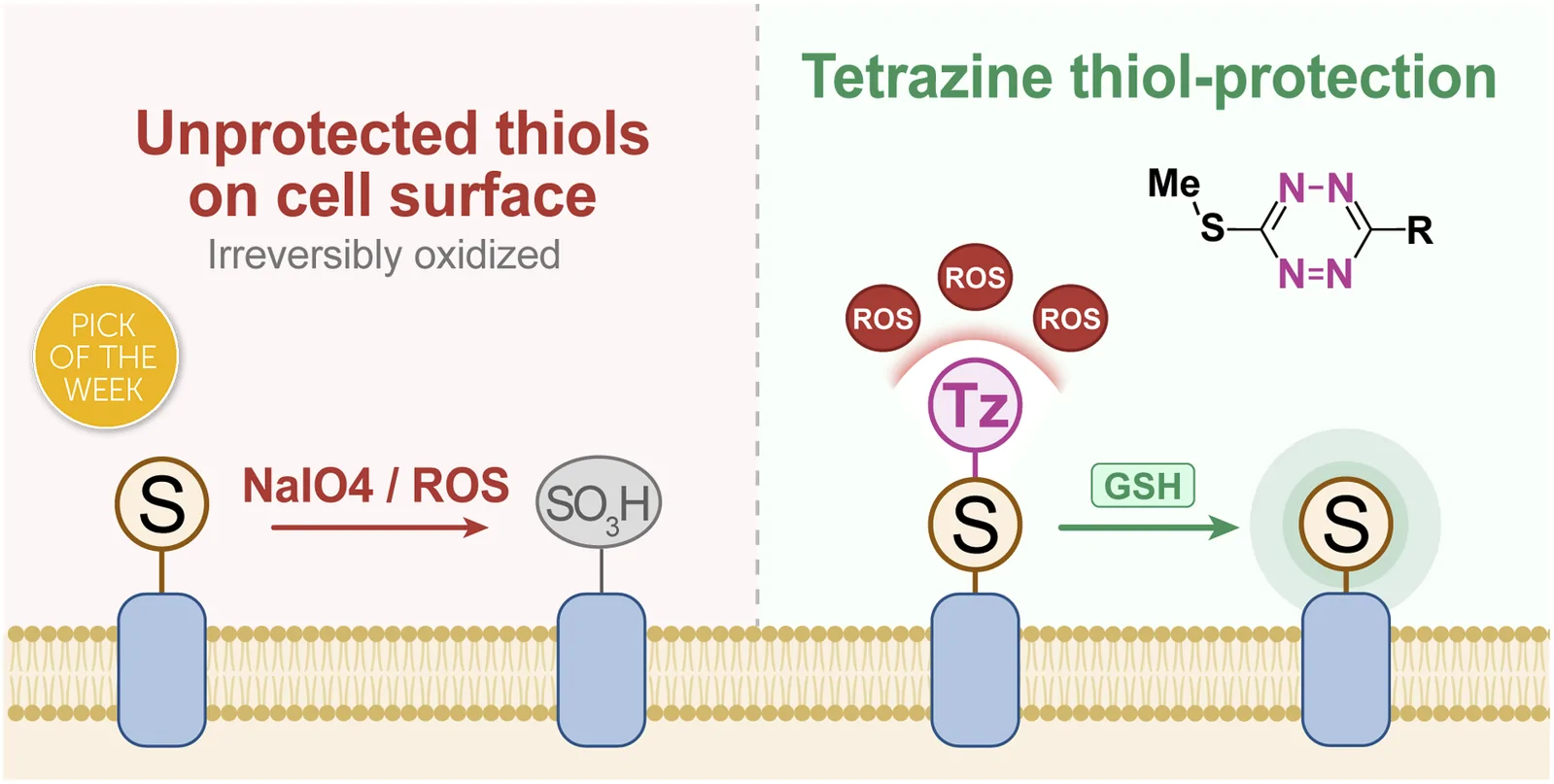

## Introduction

Cysteine residues exposed on the cell surface play critical roles in maintaining protein structure, regulating enzymatic activity, and mediating cellular signaling pathways.^1–3^ Together with low-molecular-weight thiols, these exofacial cysteines are essential for preserving redox homeostasis and protecting cells from oxidative stress.^4^ However, their high reactivity makes them particularly vulnerable to oxidation. Under oxidative or inflammatory conditions, reactive oxygen and nitrogen species readily convert thiols into disulfides or higher oxidation states such as sulfenic, sulfinic, or sulfonic acids.^2,5^ Such irreversible modifications can impair protein function, disrupt membrane integrity, and ultimately compromise cell viability.^6–8^ Beyond pathological conditions, thiol oxidation is also a recurring complication in many widely used cell-surface engineering strategies. Numerous chemical biology approaches rely on oxidative reagents to introduce reactive handles on living cells.^9,10^ For instance, sodium periodate (NaIO_4_) is commonly employed to oxidize vicinal *cis*-diols in surface glycans, generating aldehydes for further conjugation.^11–13^ While effective, this oxidative treatment inevitably affects surface-exposed thiols, leading to uncontrolled disulfide formation or irreversible overoxidation. As a result, redox-sensitive proteins and thiol-dependent cellular functions can be unintentionally disrupted. Although many surface cysteines exist as disulfides, protecting the subpopulation of functional free thiols, including those intentionally liberated *via* pre-reduction for subsequent modification, is indispensable for maintaining cellular integrity during such oxidative challenges. This intrinsic lack of chemoselectivity highlights a persistent challenge in cell-surface chemistry: how to perform oxidative modifications while preserving the integrity and functionality of surface thiols. Temporarily masking these thiols could shield them from irreversible damage.

In synthetic organic chemistry, protecting groups are routinely used to temporarily mask reactive functional groups to enable selective transformations in complex multistep syntheses.^14,15^ An effective protecting group should be easy to install and remove, stable under the intended reaction conditions, and orthogonal to other functionalities.^16^ Despite their wide utility in chemical synthesis, analogous protection strategies have rarely been translated to a live-cell context, where biocompatibility, reversibility, and temporal control are essential requirements.^17,18^ Other cysteine-masking chemistries provide useful points of comparison. DTNB can reversibly mask thiols by forming mixed disulfides and has been used to block exofacial thiols. However, these adducts remain susceptible to further thiol–disulfide exchange, making their stability dependent on the surrounding redox environment.^19,20^ By contrast, aromatic SNAr reagents developed for cysteine conjugation, including perfluoroaryl and fluorobenzene probes, generally form stable aryl thioethers and are therefore operationally irreversible under physiological conditions.^21–23^ Thiomethyl-bearing tetrazines have previously been explored as reversible thiol-reactive motifs under aqueous conditions and in peptide modification.^24,25^ However, these studies focused on molecular reactivity and conjugation rather than the temporary protection of native thiols in living systems. We therefore asked whether tetrazine–thiol exchange could be applied as a bio-protection strategy for exofacial cysteines on living cells.

Here, we report a reversible and biocompatible thiol protection strategy based on sulfide tetrazines that covalently mask cell-surface thiols *via* nucleophilic aromatic substitution (SNAr) (Fig. 1). The resulting *S*-tetrazine adducts render surface thiols inert toward oxidative damage, as demonstrated on synthetic polymeric membranes and live Jurkat and HeLa cells subjected to sodium periodate treatment. Notably, this protection can be readily reversed under physiological conditions by extracellular glutathione (GSH), restoring the native thiols through a second SNAr process. To our knowledge, this work represents the first demonstration of reversible, protecting-group-like masking of thiols directly on live-cell surfaces. By introducing the concept of “bio-protection”, this strategy extends the toolbox of bioorthogonal chemistry toward conditional control of thiol reactivity, opening new avenues for dynamic cell-surface engineering, redox biology, and thiol-dependent bioconjugation in complex biological environments.

**Fig. 1:**
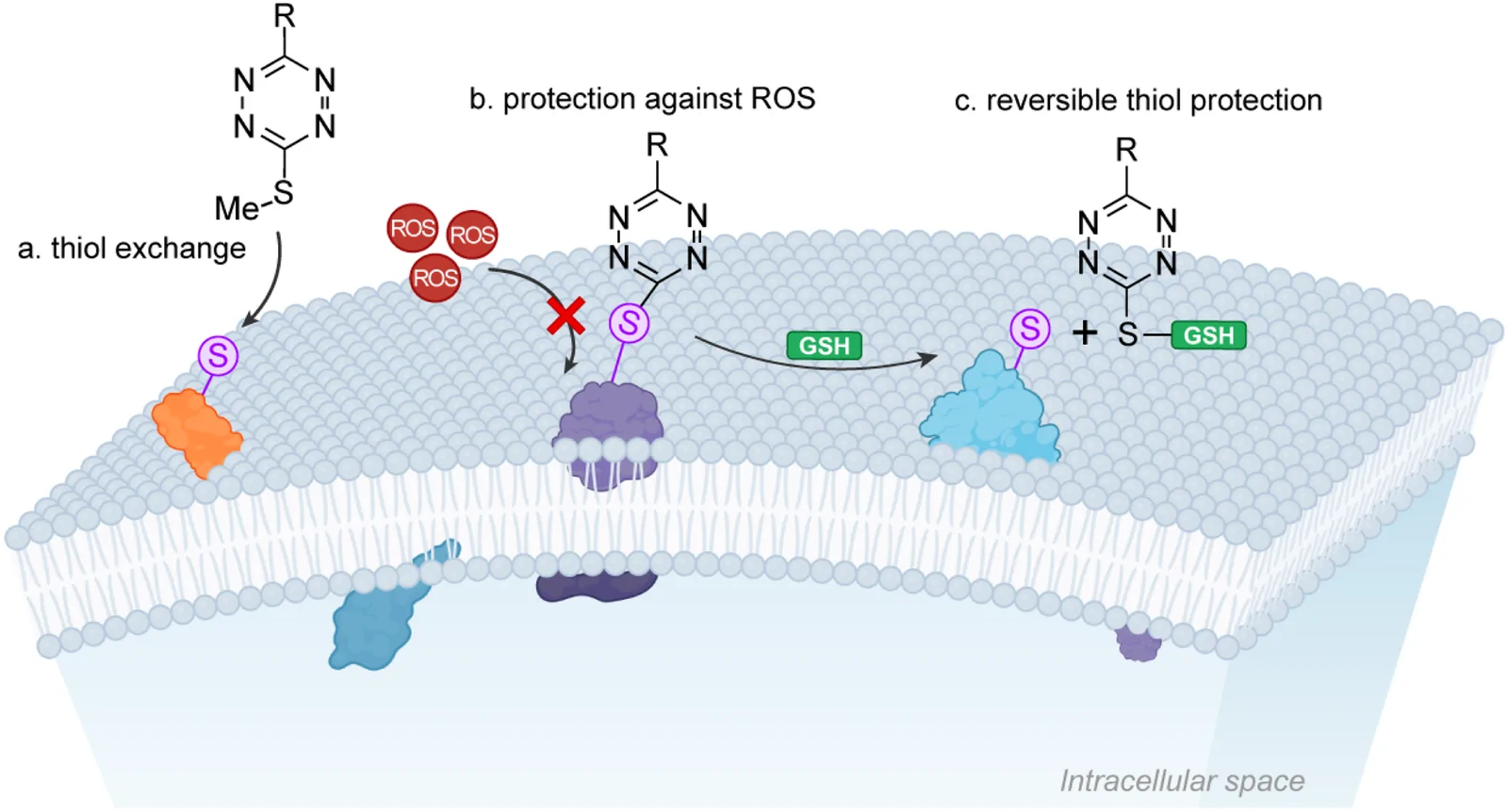
Tetrazine-based thiol protection on the cell surface. (a) Thiomethyl-bearing tetrazine reacts with cellsurface thiols to form inert *S*-tetrazine adducts. (b) Protected thiols are not affected by the oxidant represented by NaIO_4_. (c) The protected thiols are restored to native thiols through an S_N_Ar with GSH.

## Results and discussion

### Tetrazine-mediated protection of artificial cell membranes

To evaluate the effectiveness of tetrazines as protecting groups for cellmembrane-exposed thiols, we examined whether the conversion into *S*-tetrazine adducts could prevent thiol oxidation to sulfenic, sulfinic, or sulfonic acids (Fig. 2a). To initially examine the stability of tetrazine against oxidation, we exposed Tz1 and Tz2 to *N*-chlorosuccinimide, a potent electrophilic oxidant. No oxidation was observed, as identical peaks with the same retention time were observed in the HPLC trace (Fig. S1). Next, we introduced a model system to establish a suitable workflow in which surface-exposed thiols are quantitatively masked by tetrazines. For this purpose, we employed a polymeric membrane model composed of the block copolymer poly(ethylene glycol)_44_-*b*-poly(styrene)_180_ (PEG-PS) (Fig. 2b). In this system, 5% of the polymers displayed a thiol group, which was exposed on the surface of the polymeric membrane upon self-assembly into polymersomes (Fig. S2). Surface-exposed thiols of polymersomes were reacted with varying concentrations of Tz1 or Tz2 (10 nM to 100 µM), followed by labeling of unreacted thiols using fluorescein-5-maleimide (5-FM). After removal of excess fluorophore, fluorescence measurements of the polymersome suspensions enabled quantification of residual unprotected thiols. As shown in Fig. 2c, both Tz1 and Tz2 effectively masked surface thiols in a dose-dependent manner up to 50 µM. We observed saturation at concentrations higher than 50 µM. The gradual reduction in fluorescence intensity for both tetrazines indicated comparable efficiency in binding to free thiols on the polymeric membrane. To control for non-specific 5-FM adsorption, thiol-free polymersomes incubated with 5-FM were measured separately (red line), confirming the background signal. At higher tetrazine concentrations (50–100 µM), similar fluorescence was detected for both thiol-containing and thiol-free polymersomes, suggesting complete thiol masking. This interaction was further validated by quantifying tetrazine consumption *via* HPLC (Fig. S3). To assess the reversibility of tetrazine protection, we introduced glutathione (GSH) to the system, as it is a non-toxic endogenous antioxidant. We hypothesized that masking thiols as electron-deficient sulfide tetrazines would prevent their irreversible oxidation during cellsurface engineering. Specifically, this strategy aims to shield thiols from sodium periodate (NaIO_4_), a common reagent for generating aldehyde handles that otherwise converts free thiols into non-nucleophilic oxoacids (*e.g.*, sulfenic, sulfinic, or sulfonic acids). Following tetrazine treatment, polymersomes were exposed to NaIO_4_, then to GSH, and finally labeled with 5-FM (Fig. 2d). Fluorescence recovery (Fig. 2e) was significantly higher in Tz1- and Tz2-treated samples compared to unprotected controls, supporting the hypothesis that tetrazine masking protects surface thiols from oxidation and can be reversed by GSH. HPLC further confirmed the formation of GSH–tetrazine conjugates (Fig. S4).

**Fig. 2:**
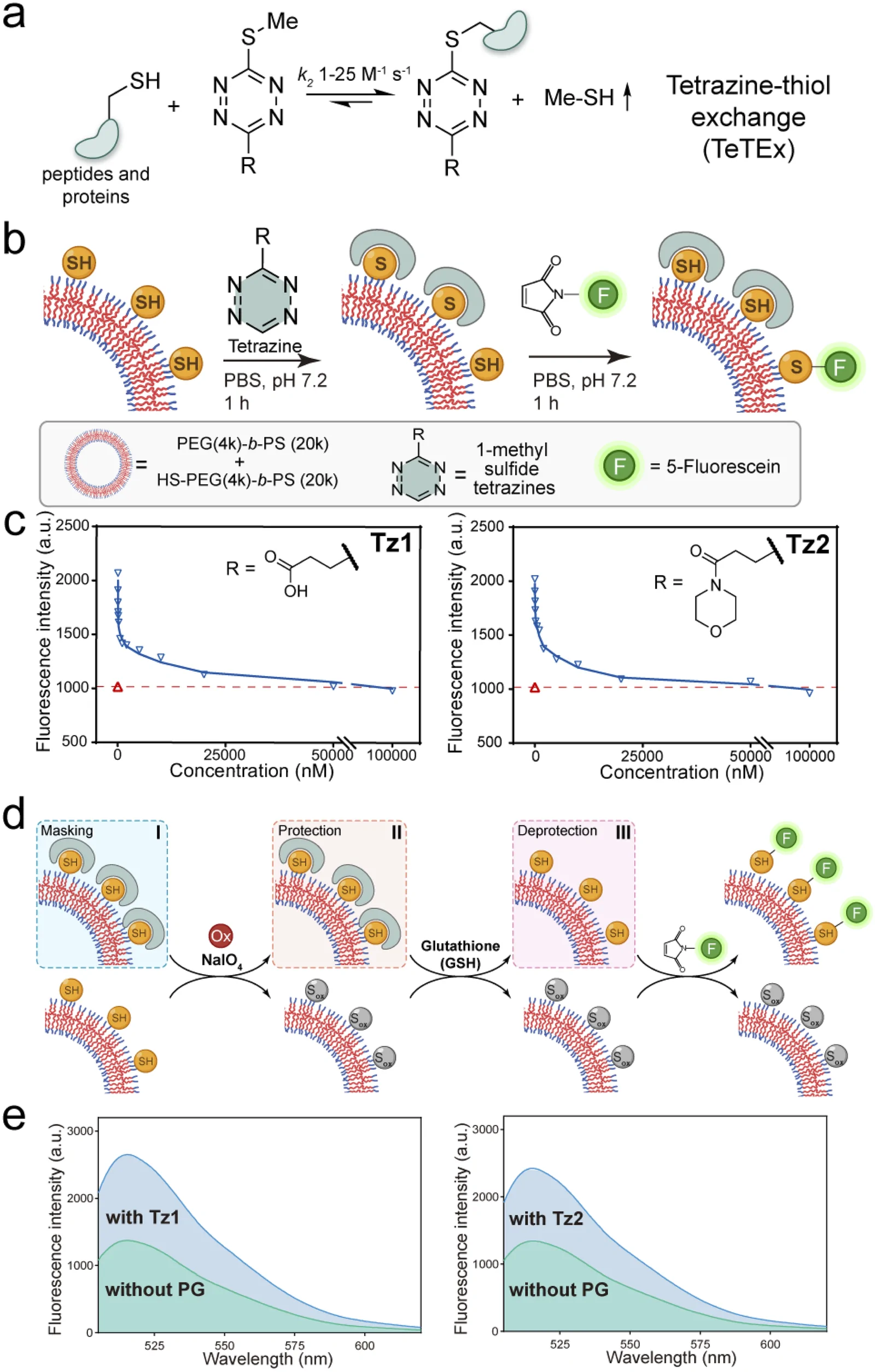
Thiol protection on polymeric membranes. (a) Tetrazines react with thiols in proteins or peptides *via* S_N_Ar. (b) Schematic illustration of thiol protection on polymersomes: tetrazines covalently bind to surface-exposed thiols, followed by fluorescent labeling of unreacted thiols using 5-FM. (c) Fluorescence intensity of polymersomes treated with increasing concentrations of tetrazine (10 nM to 100 µM). High concentrations of tetrazine result in higher levels of thiol masking, as reflected by reduced fluorescence intensity. (d) Schematic overview of the reversible thiol protection under oxidative conditions. Protected thiols resist oxidation and are revealed *via* 5-FM labeling after deprotection. (e) Fluorescence spectra of surface thiols labeled with 5-FM after sequential treatment with tetrazines, oxidation, and GSH.

### Tetrazine-mediated bio-protection demonstrated on live cells

To assess whether tetrazine chemistry can effectively protect native cellsurface thiols, we evaluated the reactivity of Tz1 and Tz2 with exofacial cysteine residues on live cells. Jurkat and HeLa cells were selected to represent two distinct cellular models. Jurkat cells are a human T-lymphocyte-derived suspension cell line, and surface thiols are well documented on lymphocytes and are involved in redox-sensitive cellular functions.^26^ HeLa cells were included as an adherent epithelial cancer-cell model, in which exofacial thiol-dependent processes have also been demonstrated.^27^ To simulate a scenario where high-density surface thiols are liberated for downstream functionalization, we initially treated the cells with tris(2-carboxyethyl)phosphine (TCEP), a mild and water-soluble reducing agent that selectively reduces disulfide bonds without disrupting membrane integrity.^28,29^ Then, cells were incubated with varying concentrations of Tz1 or Tz2 at 37 °C for 2 hours (concentrations: 10 nM, 500 nM, 2 µM, and 100 µM), allowing covalent coupling to form a stable *S*-tetrazine adduct, followed by treatment with NaIO_4_ and GSH to reverse the protection. The remaining unmasked thiols were then fluorescently labeled using fluorescein-5-maleimide (5-FM, 2 µM), enabling quantification of thiol restoration *via* flow cytometry (Fig. 3a). Similar to the observation on the polymeric membrane, flow cytometry analysis showed a dose-dependent increase in fluorescence signal intensity, with higher concentrations of initial tetrazine resulting in enhanced fluorescence signal when labelled with 5-FM (Fig. 3b, c, S6–S9 and S13), indicating that higher tetrazine concentrations preserved a larger fraction of cell-surface thiols for fluorescent labelling after deprotection. To evaluate the possible contribution of residual thiols that were neither masked nor oxidized, we performed an additional control without GSH-mediated deprotection. Cells treated with 100 µM Tz2 without GSH showed fluorescence comparable to the 0 nM tetrazine control, suggesting that such residual thiols contributed only minimally to the final 5-FM signal (Fig. S14). At low tetrazine concentrations (10 nM), Jurkat cells exhibited a significant increase in fluorescence for both Tz1 and Tz2 compared with untreated cells (Tz1: 305.0 ± 7.9 *vs.* 188.0 ± 62.0, 10 nM *vs.* 0, *p* = 0.032; Tz2: 792.0 ± 11.8 *vs.* 649.3 ± 0.6, 10 nM *vs.* 0, *p* < 0.001). In HeLa cells, the response at 10 nM was less consistent: Tz1 treatment did not result in a statistically significant increase in fluorescence (1120.7 ± 101.0 *vs.* 1008.7 ± 25.5, *p* = 0.136), whereas Tz2 treatment showed a significant increase (765.7 ± 8.4 *vs.* 666.7 ± 5.0, *p* < 0.001). These results indicate that tetrazine-mediated thiol masking at low concentration is both cell-type- and tetrazine-dependent, while more robust protection requires higher tetrazine concentrations. Importantly, the observed recovery of fluorescence upon GSH treatment confirms that oxidation by NaIO_4_ can be effectively mitigated by prior tetrazine labeling.

**Fig. 3:**
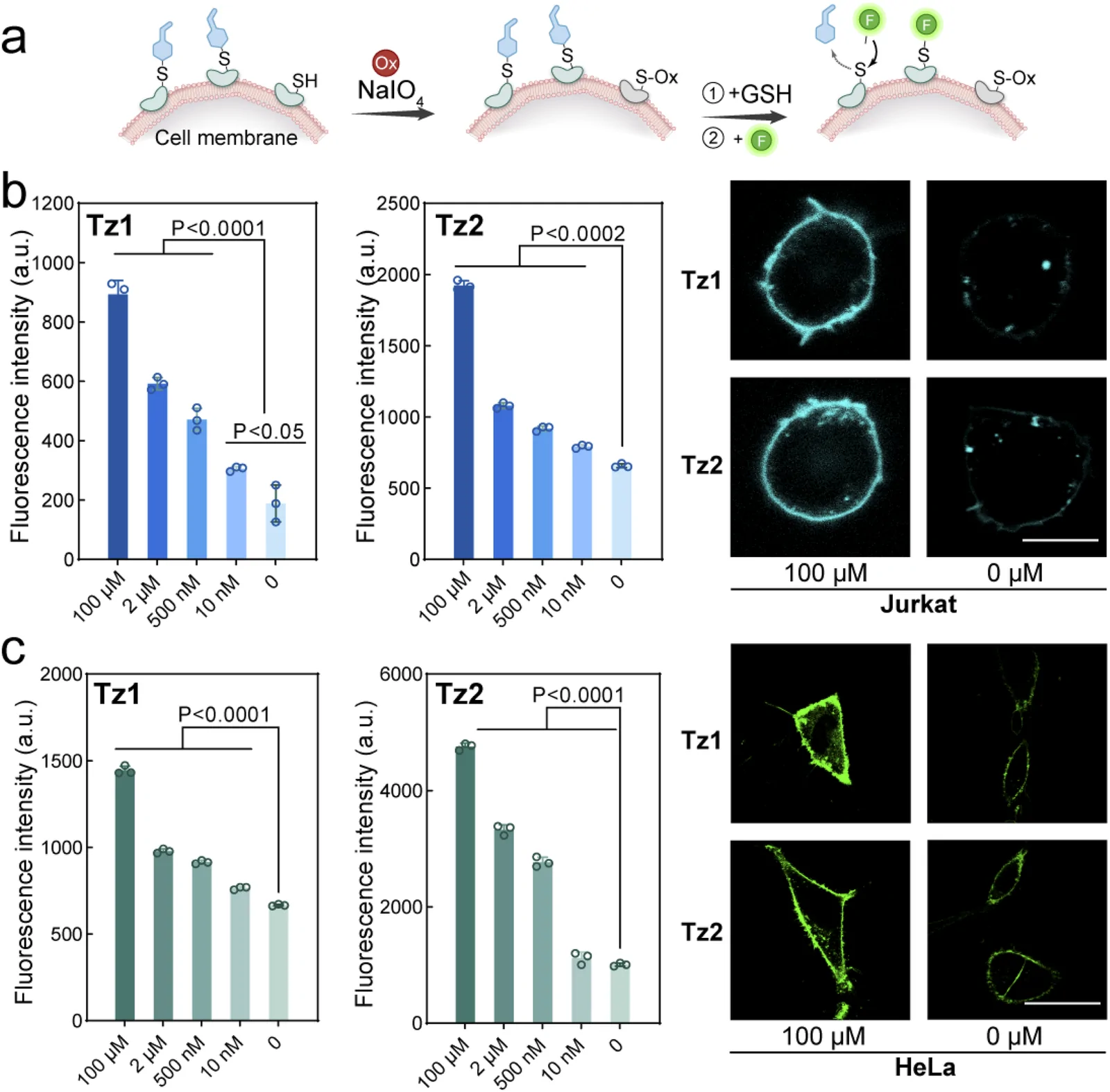
Thiol protection on cell surfaces. (a) Schematic illustration of tetrazine-mediated protection of cell-surface thiols from NaIO_4_ oxidation, followed by GSH-mediated removal of the tetrazine groups and fluorescent labelling of the restored thiols with 5-FM. (b) Flow cytometry and confocal analysis of fluorescence intensities in Jurkat cells following treatment with various concentrations of tetrazines (*n* = 3, scale bar = 10 µm). (c) Flow cytometry and confocal analysis of fluorescence intensities in HeLa cells following treatment with various concentrations of tetrazines (*n* = 3, scale bar = 10 µm). Statistical significance was assessed using ordinary one-way ANOVA followed by Dunnett's multiple-comparisons test.

### Bio-protection of tetrazine preserves thiol-mediated cellular uptake

Thiol-mediated cellular uptake was first described by Ryser's group in the 1990s.^30^ Since then, numerous studies have demonstrated that free thiol groups on the cell surface facilitate enhanced uptake of thiol-reactive cargo, including peptides, nanoparticles, and other entities.^31–33^ This mechanism, driven by dynamic disulfide exchange or thiol–maleimide interactions, has been widely exploited to improve the delivery efficiency of functional materials across biological membranes.^34^ To ensure that the thiol-masking process is restricted to the cell surface and avoids interference with intracellular redox homeostasis, we selected the carboxylated derivative Tz1 for thiol-mediated uptake experiments. The presence of the charged carboxylate moiety at pH 7.4 minimizes passive membrane permeability, a crucial feature for maintaining the integrity of the internal thiol pool (*e.g.*, GSH).^35^ Given that oxidants are frequently used in cellsurface engineering protocols-for example, to introduce aldehyde groups *via* periodate oxidation-we aimed to investigate how such oxidative treatment affects thiol-dependent uptake and whether tetrazine-based thiol protection can mitigate these effects. For this uptake experiment, NIH-3T3 cells were used as an adherent cell model because adherent cells are more suitable for confocal z-stack imaging and single-cell analysis. This also enabled comparison between two distinct adherent cell models. Therefore, in this experiment (as illustrated in Fig. 4a), HeLa and NIH-3T3 cells were first incubated with tetrazine for 4 hours to form stable *S*-tetrazine adducts. Then the cells were exposed to NaIO_4_ to simulate an oxidizing environment, followed by incubation with 2 mM GSH to remove the tetrazine protection (Tz + NaIO_4_ + GSH). Finally, fluorescence-labeled thiol-displaying polymersomes were incubated with the cells. As shown in the fluorescence images in Fig. 4c, the PBS control group and the Tz + NaIO_4_ + GSH group exhibited comparable levels of polymersome uptake. This indicates that tetrazine masking protected cell-surface thiols from oxidation during NaIO_4_ treatment and that subsequent GSH-mediated deprotection restored their reactivity. Consequently, the cells retained a level of thiol-mediated uptake comparable to that of untreated cells.

**Fig. 4:**
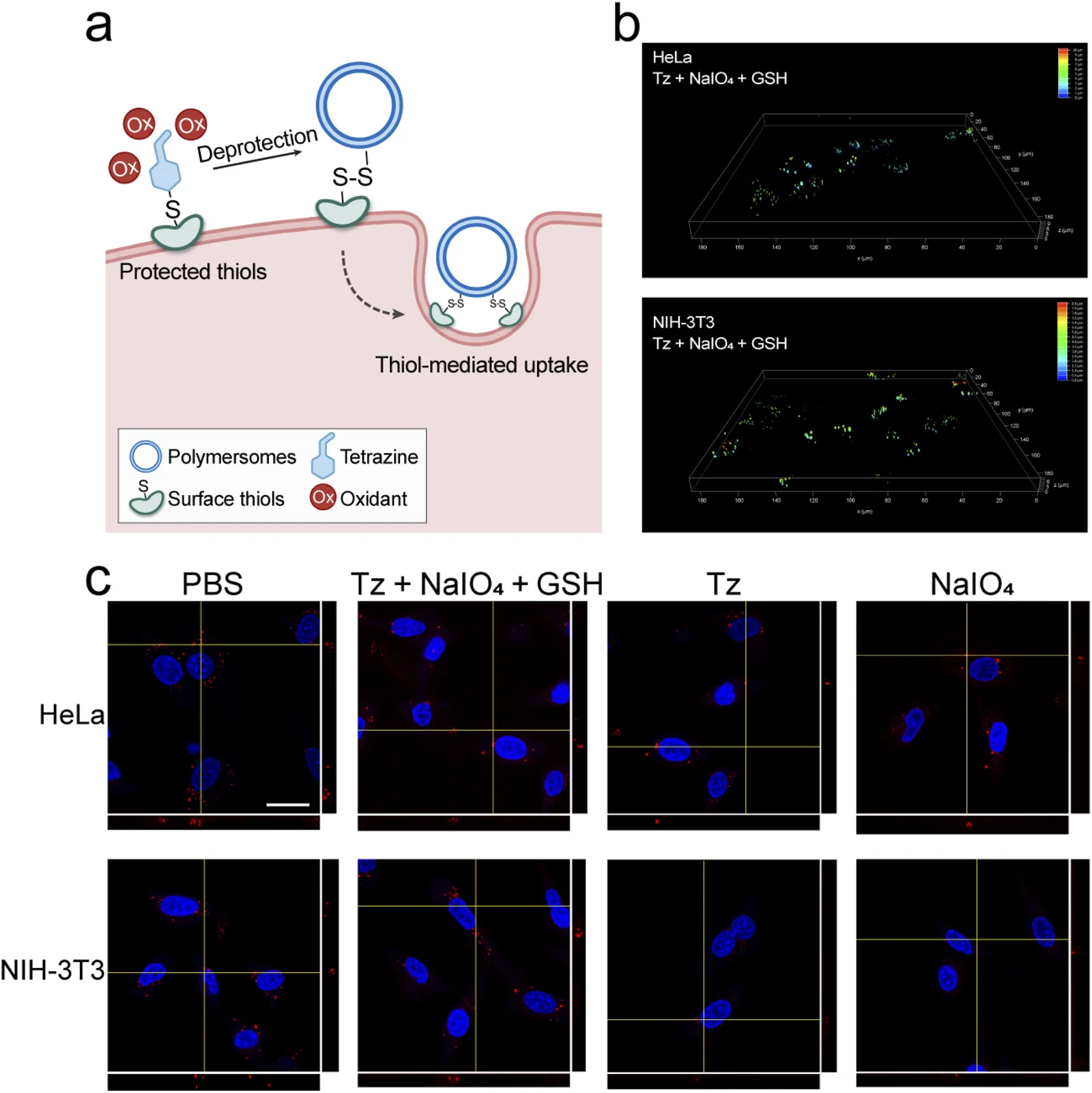
Thiol-mediated cellular uptake. (a) Schematic illustration of the reversible masking of cell-surface thiols and their subsequent availability for thiol-mediated uptake following deprotection. (b) Three-dimensional reconstructions of the confocal z-stacks showing the spatial distribution of polymersome-associated fluorescence. (c) Representative orthogonal confocal z-stack images of HeLa and NIH-3T3 cells following the indicated treatments and incubation with Nile Red-labelled thiol-functionalized polymersomes. Cell nuclei were stained with DAPI and are shown in blue, whereas Nile Red fluorescence from the polymersomes is shown in red.

In contrast, cells treated solely with NaIO_4_ (the NaIO_4_ group) showed significantly reduced uptake, suggesting that NaIO_4_ severely affected cell function by oxidizing surface thiols, rendering them unreactive. Similarly, a low level of cellular uptake was also observed when cells were treated only with tetrazine but without subsequent deprotection (the Tz group), as the tetrazine reacted with surface thiols, thereby inhibiting the interaction between surface thiols and thiols on the polymersome surface. Three-dimensional fluorescence reconstructions in Fig. 4b, S10 and S11 visualize the spatial distribution of polymersome-associated fluorescence throughout the confocal z-stacks.

## Conclusions

In summary, we have developed a reversible, biocompatible strategy to protect cell-surface-exposed cysteine thiols using 3,6-disubstituted tetrazines. This “bio-protection” approach enables the rapid and chemoselective masking of surface thiols, rendering them inert to oxidative damage while maintaining cellular viability. Importantly, the protection is readily reversed under physiological conditions by glutathione, restoring native thiols through a nucleophilic aromatic substitution mechanism. We demonstrated the efficiency of this strategy on both synthetic polymeric membranes and live Jurkat and HeLa cells, showing that protected thiols are shielded from NaIO_4_-induced oxidation and remain functional for thiol-mediated processes such as cellular uptake. To our knowledge, this is the first demonstration of a protecting-group-like strategy that transiently masks native exofacial thiols on live cells, preserves them during oxidative cell-surface engineering, and restores their thiol-mediated function upon GSH-triggered deprotection. This strategy expands the chemical toolbox for dynamic cell-surface engineering and paves the way for precisely controlling thiol-dependent cellular processes, with broad implications for redox biology, targeted delivery systems, and the design of advanced biointerfaces.

## Author contributions

M. S., K. N., and D. A. W. conceived the project and designed the experiments. K. G. carried out the chemical synthesis. M. S., C. W., and J. S. performed the polymersome characterization and HPLC analysis. M. S. and C. W. conducted the *in vitro* and live-cell experiments. Data analysis was jointly performed by M. S., C. W., and J. S. The manuscript was drafted by M. S. and revised and edited by all authors. K. N. and D. A. W. supervised the research.

## Conflicts of interest

The authors declare no competing interests.

## Supplementary Material

SC-OLF-D6SC04306B-s001

## Data Availability

The data supporting this article have been included as part of the supplementary information (SI). Supplementary information: NMR spectra, HPLC spectra, individual flow cytometry data, and further experimental details. See DOI: https://doi.org/10.1039/d6sc04306b.
