# Supplementary material for: Tetrazine-based bio-protection: reversible masking of cell surface thiols for orthogonal cellular chemistry
Source: Chem Sci. 2026 Nov 3. Online ahead of print. doi: 10.1039/d6sc04306b (PMC13730538; doi:10.1039/d6sc04306b)
Supplement: Supplementary file 1 [file SC-OLF-D6SC04306B-s001.pdf]

Supplementary Information (SI) for Chemical Science.
This journal is © The Royal Society of Chemistry

Tetrazine-Based Bio-Protection: Reversible Masking of Cell Surface Thiols for Orthogonal Cellular Chemistry

Mingchen Sun, [1] Katerina Gavriel, [1] Chenxuan Wan, [1] Jelte Schermer, [1] Kevin Neumann, [1,*] and Daniela A. Wilson [1,*]

[1]Radboud University Nijmegen, Institute for Molecules and Materials, Heyendaalseweg 135, 6525 AJ, Nijmegen, The Netherlands

[*]Corresponding author e-mail: d.wilson@science.ru.nl, kevin.neumann@ru.nl

Content

Materials and methods 2
1. Chemicals 2
2. General instrumentation 2
3. Synthesis 3
   Methyl thiocarbohydrazide hydrogen iodide salt (1) 3
   tert-Butyl [(3-methyloxetan-3-yl)methyl]succinate (2) 3
   3-methylthio-6-(3-tert-butylcarboxypropyl)-1,2,4,5-tetrazine (3) 3
   3-methylthio-6-(3-carboxypropyl)-1,2,4,5-tetrazine (4) 4
   3-methylthio-6-(3-morpholin-4-ylpropan-2-one)-1,2,4,5-tetrazine (5) 4
4. Preparation of polymersomes 5
5. Thiol protection on the polymersome surface 5
6. Reversible thiol protection on the polymersome surface 5
7. High-performance liquid chromatography (HPLC) 6
8. Visualization of thiol protection on giant polymersomes 6
9. Cell culture 6
10. Cytotoxicity 6
11. Thiol protection on the cell surface 7
12. Thiol-mediated cellular uptake 7
13. Kinetic analysis of tetrazine-mediated protection and GSH-mediated deprotection 7
14. Statistical analysis 8
Supplementary figures 9
Synthesis 27

Materials and methods

1. Chemicals

Reagents were obtained from Sigma Aldrich/Merck (Zwijndrecht, The Netherlands), Fluorochem BV (Amsterdam, The Netherlands) and TCI Europe (Zwijndrecht, Belgium). The reagents were used without purification unless otherwise stated. Solvents were obtained from VWR, Fisher, Acros Organic and Sigma Aldrich/Merck. Solvents were dried by passing over activated alumina columns in an MBraun MB SPS800 under a nitrogen atmosphere and stored under nitrogen. Reactions were carried out in air unless stated otherwise. Air-sensitive reactions were carried out under an atmosphere of nitrogen using the Schlenk technique. Reactions and fractions from flash column chromatography were monitored by thin-layer chromatography using glass TLC plates (Merck, TLC Silica gel 60 F254) and if necessary visualized by staining with potassium permanganate or ninhydrin solution. Column chromatography was performed on VWR SiO2 Type (40-63 mesh) using a forced flow of air at 0.5-1.0 bar.

2. General instrumentation

Nuclear Magnetic resonance (NMR) characterization was carried out on a Bruker AVANCE HD nanobay console with a 9.4 T Ascend magnet (400 MHz) and a Bruker AVANCE III console with an 11.7 T UltraShield Plus magnet (500 MHz) equipped with a Bruker Prodigy cryoprobe, in chloroform ($CDCl_3$) or DMSO-d_6. NMR spectra were recorded at 298 K unless otherwise stated. Tetramethylsilane (TMS, δ 0.00 ppm) was used as an internal standard for ^{1}H NMR. Chemical shifts are reported in parts per million (ppm) and coupling constants are reported as J values in Hz. Peak assignment is based on 2D COSY, $^{1}H-^{13}C$ HSQC, and $^{1}H-^{13}C$ HMBC spectra. The splitting patterns are indicated as follows: s, singlet; br. s, broad singlet; d, doublet; t, triplet; m, multiplet. Mass spectra were measured with JEOL AccuTOF CS JMST100CS.

3. Synthesis

Methyl thiocarbohydrazide hydrogen iodide salt (1)

The salt was prepared following a reported protocol.1 A solution of thiocarbohydrazide (5.0 g, 47.1 mmol, 1 eq.) and methyl iodide (3.2 mL, 51.8 mmol, 1.1 eq.) in ethanol (0.3 M) was added to a dried round-bottomed flask. The solution was refluxed at 80°C for 3 h and cooled to room temperature. The reaction mixture was diluted with heptane and cooled to -20°C overnight. The resulting solid was collected via filtration, washed with a cold ethanol/heptane (1:1 v/v) mixture, and dried under reduced pressure. The product was obtained as a white solid (8.9 g, 76% yield), and the spectroscopic data were in agreement with literature data. ^{1}H NMR (400 MHz, DMSO) δ 9.62 (s, 1H), 5.15 (s, 2H), 3.33 (s, 1H), 2.39 (s, 3H). MS (*m/z*): calcd for $[C_4H_{18}IN_8S_2]^+$: 369.27, found: 369.02 $[2M+I]^+$.

tert-Butyl [(3-methyloxetan-3-yl)methyl]succinate (2)

3-methyl-3-oxetanemethanol (3.2 g, 31.6 mmol, 1.1 eq.), EDC·HCl (6.6 g, 34.4 mmol, 1.2 eq.) and DMAP (0.4 g, 2.9 mmol, 0.1 eq.) were added in a dry flask under an inert atmosphere. Anhydrous DCM (0.5 M) was added to the flask, and the solution was cooled to 0°C. Subsequently, tert-butyl hemisuccinate (5.0 g, 28.7 mmol, 1.0 eq.) was added, and the solution was stirred at 0°C for 15 min under nitrogen and at room temperature overnight. Then, the reaction mixture was diluted with DCM and washed with saturated sodium bicarbonate solution, water, and brine. The organic layer was combined, dried over sodium sulfate, and concentrated in vacuo. The residue was purified by flash chromatography (30% Acetone in DCM), yielding a colorless oil (7.3 g, 98% yield). ^{1}H NMR (400 MHz, $CDCl_3$) δ 4.50 (d, J = 6.0 Hz, 1H), 4.37 (d, J = 6.0 Hz, 1H), 4.18 (s, 1H), 2.61 (ddd, J = 7.0, 5.7, 1.6 Hz, 1H), 2.58 – 2.50 (m, 1H), 1.43 (s, 4H), 1.32 (s, 1H).^{13}C NMR (101 MHz, $CDCl_3$) δ 172.7, 171.5, 80.9, 79.7, 69.0, 39.2, 30.4, 29.3, 28.2, 21.3. HRMS (*m/z*): calcd for $[C_{13}H_{22}O_5Na]^+$: 281.3006, found: 281.1350 $[M+Na]^+$.

3-methylthio-6-(3-tert-butylcarboxypropyl)-1,2,4,5-tetrazine (3)

In a dried round-bottomed flask, oxetane ester 2 (7.0 g, 27.1 mmol, 1.2 eq.) was dissolved in anhydrous DCM (1.0 M). The solution was cooled to -12 °C and boron trifluoride etherate (3.3 mL, 27.1 mmol, 1.2 eq.) was added dropwise via syringe. The mixture was stirred for 4 hours

under nitrogen while cooled at -12°C, never allowing the temperature to go above -4°C. The reaction was monitored by TLC of aliquots that were quenched with trimethylamine before spotting the TLC plate. Once the reaction was complete, the reaction mixture was quenched with pyridine (4.6 mL, 56.6 mmol, 2.5 eq.), and then a solution of salt 1 (5.6 g, 22.6 mmol, 1.0 eq.) in DMF (1.0 M) was added dropwise via syringe. The mixture was stirred vigorously, and a nitrogen flow was applied to remove the DCM. The resulting mixture was allowed to stir for 30 min under nitrogen at 80°C. After cooling to room temperature, PIDA (7.3 g, 22.6 mmol, 1.0 eq.) was added to the flask and the mixture was allowed to stir for 30 min at room temperature. The mixture was diluted with DCM and sequentially washed with saturated sodium bicarbonate, water, and brine, dried over magnesium sulfate, and concentrated under reduced pressure. The residue was purified by flash column chromatography on silica gel (20-50% EtOAc in heptane), yielding a pink oil (3.6 g, 52% yield). ^{1}H NMR (400 MHz, $CDCl_3$) δ 3.51 (t, J = 7.1 Hz, 2H), 2.91 (t, J = 7.1 Hz, 2H), 2.72 (s, 3H), 1.41 (s, 9H). ^{13}C NMR (101 MHz, $CDCl_3$) δ 175.7, 171.3, 166.8, 81.2, 32.4, 29.7, 28.2, 13.5. MS (*m/z*): calcd for $[C_{10}H_{16}N_4O_2SNa]^+$: 279.31, found: 279.13 $[M+Na]^+$.

3-methylthio-6-(3-carboxypropyl)-1,2,4,5-tetrazine (4)

Protected tetrazine 3 (3.6 g, 14.0 mmol, 1.0 eq.) was dissolved in 15 mL of a 4:1 DCM/TFA mixture (v/v) at 0°C. Then, the mixture was stirred at room temperature for 18 hours. The crude mixture was concentrated by applying a flow of N_2. Then the crude was purified by column chromatography (50-100% EtOAc in heptane), yielding a pink oil (2.0 g, 73% yield). ^{1}H NMR (400 MHz, $CDCl_3$) δ 3.58 (t, J = 7.0 Hz, 2H), 3.08 (t, J = 7.1 Hz, 2H), 2.74 (s, 3H). ^{13}C NMR (101 MHz, $CDCl_3$) δ 177.0, 176.0, 166.1, 30.5, 29.1, 13.5. MS (*m/z*): calcd for: $[C_6H_9N_4O_2S]^+$: 201.23, found: 201.09 $[M+H]^+$.

3-methylthio-6-(3-morpholin-4-ylpropan-2-one)-1,2,4,5-tetrazine (5)

HBTU (104 mg, 0.3 mmol, 1.1 eq.) was added to a solution of tetrazine **4** (50 mg, 0.3 mmol, 1.0 eq.) in anhydrous DMF (0.2 M). DIPEA (87 µL, 0.5 mmol, 2.0 eq.) was added, and the mixture was left to react for 15 min. Morpholine (24 µL, 0.3 mmol, 1.1 eq.) was added, and the mixture was stirred for 40 minutes at room temperature. The reaction mixture was diluted with DCM and washed with water (5x) and brine. The organic layer was dried over magnesium sulfate, filtered, and concentrated under reduced pressure. The crude was purified by column

chromatography (2% MeOH in DCM), yielding a pink oil (46 mg, 68% yield). ^{1}H NMR (400 MHz, $CDCl_3$) δ 3.74 – 3.50 (m, 10H), 3.04 (t, J = 6.9 Hz, 2H), 2.72 (s, 3H). ^{13}C NMR (101 MHz, $CDCl_3$) δ 175.6, 169.7, 167.0, 67.0, 66.7, 45.9, 42.2, 29.7, 29.6, 13.5. HRMS (*m/z*): calcd for $[C_{10}H_{16}N_5O_2S]^+$: 270.1019, found: 270.1011 $[M+H]^+$.

4. Preparation of polymersomes

9.5 mg of PEG_{44}-*b*-PS_{180} and 0.5 mg of SH-PEG_{44}-*b*-PS_{180} were dissolved in a 1 mL mixture of tetrahydrofuran (THF) and dioxane (4:1, v/v). After stirring for 30 min, 1 mL of Milli-Q water was added to the solution at a rate of 1 mL/h at room temperature under vigorous stirring. Afterwards, approximately 2.5 mL of Milli-Q water was promptly mixed with the suspension to quench the structure. The organic solvent was removed by repeated centrifugation. For the fluorescence-labelled polymersomes used in Figure 4, the preparation followed the same protocol, except 20 µL of Nile Red solution in THF (2 mg/mL) was added to the THF/dioxane mixture.

5. Thiol protection on the polymersome surface

To test the tetrazine stability under oxidative conditions, **Tz1** and **Tz2** were dissolved in PBS (2 µM) and mixed with N-chlorosuccinimide (10 µM). After 30 min of incubation at 37°C, the mixture was then diluted with methanol and detected by reverse-phase HPLC (Agilent AG1120) with a Phenomenex PRODIGY ODS-3 column (5 µm, 100 Å, 250 × 4.60 mm) and UV absorbance (215 and 254 nm). Eluent: 0.1% formic acid in Milli-Q and ACN (5- 95% ACN in Milli-Q over 25 min).

100 µL of polymersome suspension (1 mg/mL, calculated by the PEG-PS) was mixed with the solutions of **Tz1** or **Tz2**. The final concentrations of **Tz1** and **Tz2** were: 10 nM, 20 nM, 50 nM, 100 nM, 200 nM, 500 nM, 1 µM, 2 µM, 5 µM, 10 µM, 20 µM, 50 µM and 100 µM. The mixtures were incubated at 37°C for 3 hours with the lids open, followed by centrifugation at 14000 rpm for 10 min. The sediments were resuspended with 200 µL of 5-FM (concentration: 100 µM) and incubated for another 2 hours at 37°C. Afterward, the polymersome suspensions were centrifuged (14000 rpm, 10 min) and washed with Milli-Q three times before measuring the fluorescence intensity at 519 nm (excitation wavelength: 494 nm).

6. Reversible thiol protection on the polymersome surface

100 μL of polymersome suspension (1 mg/mL, calculated by the PEG-PS) was mixed with the solutions of **Tz1** or **Tz2** with a final concentration of 50 μM and incubated at 37°C for 3 hours with the lids open. Next, the mixture was centrifuged to remove the unreacted tetrazine. $NaIO_4$ was added to the systems (final concentration: 2 mM) for another 3 hours of incubation. After removing the excess $NaIO_4$ by centrifugation, 500 μL of GSH (concentration: 5 mM) was used to resuspend the polymersomes and incubated for 3 hours. Lastly, the polymersomes were incubated with 5-FM at 37°C for 3 hours before measuring the fluorescence intensity at 519 nm (excitation wavelength: 494 nm). For the negative control, the incubation was the same except for the addition of **Tz1** or **Tz2**.

7. High-performance liquid chromatography (HPLC)

100 μL of polymersome suspension (1 mg/mL, calculated by the PEG-PS) was mixed with the solutions of **Tz1** or **Tz2**. The mixtures were incubated at 37°C for 3 hours with the lids open. Afterwards, the mixture was filtered with a 0.22 μm syringe filter and detected by reverse-phase HPLC (Agilent AG1120) with a Phenomenex PRODIGY ODS-3 column (5 μm, 100 Å, 250 × 4.60 mm) and UV absorbance (215 and 254 nm). Eluent: 0.1% formic acid in Milli-Q and ACN (5- 95% ACN in Milli-Q over 25 min). For the reversibility study, polymersomes incubated with tetrazine, $NaIO_4$, and GSH were used and detected with the same methodology.

8. Visualization of thiol protection on giant polymersomes

To prepare the giant polymersomes, 1.25 mg of PEG-*b*-PBD was dissolved in 1 mL of chloroform, and the organic solvent was removed by a direct stream of nitrogen. After desiccation in a vacuum for 4 hours, the giant polymeric vehicle was formed upon the addition of 3 mL of water and incubation at 60°C for 48 h. The obtained giant polymersome suspension was then mixed with Py-SH aqueous solutions (final concentrations: 20 μM).

9. Cell culture

All cells were purchased from the American Type Culture Collection (ATCC). HeLa cells and NIH-3T3 cells were cultured in DMEM. Jurkat cells were cultured in RPMI medium. All media contained 10% fetal bovine serum (FBS) and antibiotics (penicillin 100 U/mL and streptomycin 100 μg/mL) at 37°C with 5% CO_2. Trypsin/EDTA was used to digest cells.

10. Cytotoxicity

NIH-3T3 cells were seeded in a 96-well plate at a density of 5× 10^4 per well and incubated overnight for adherence. **Tz1** and **Tz2** were added at a series of concentrations to each well and incubated for 72 hours of incubation at 37°C. After washing with PBS and refreshing the medium, 10 µL of CCK-8 was added to each well for another 3 hours of incubation. Cell viability was measured by the Spark M10 plate reader at 450 nm.

11. Thiol protection on the cell surface

HeLa cells were seeded in an 8-well plate (ibidi GmbH) with a density of 5× 10^4 per well and incubated overnight for adherence. TCEP was added to each well to reduce the cell surface thiols for 1 hour (final concentration: 0.5 mM). After washing with PBS, cells were incubated with **Tz1** or **Tz2** (final concentration: 10 nM, 500 nM, 2 µM, and 100 µM) for 2 hours at 37°C, followed by washing with PBS and incubating with $NaIO_4$ (1.6 mM, 30 min), GSH (2 mM, 2 hours), and 5-FM (2 µM, 30 min in the dark). Confocal microscopy (SP8x AOBS-WLL, Leica) was used to observe the fluorescence of 5-FM using the FITC channel (Ex=498 nm). Flow cytometry was used to analyze the fluorescence intensity of the cells using the FITC channel. The protocol and treatment for Jurkat cells were the same. After treatment, cells were transferred to an 8-well dish with poly-L-lysine coating (ibidi) at a density of 1×10^5 cells per mL before observing by confocal microscopy.

12. Thiol-mediated cellular uptake

HeLa cells and NIH-3T3 were seeded in 8-well plates (ibidi GmbH) at a density of 5× 10^4 per well and incubated overnight to allow adherence. After refreshing the culture medium, cells were incubated with TCEP (0.5 mM) for 30 min at 37°C and then subjected to the following treatments: (a) PBS only (control); (b) Tz1 (500 nM, 4 h), followed by $NaIO_4$ (2 mM, 1 h), and GSH (2 mM, 2 h) successively; (c) Tz1 (500 nM, 4 h) alone; or (d) NaIO4 (2 mM, 1 h). Subsequently, Nile Red-labeled polymersomes were added at a polymer concentration of 300 µg/mL for 3 hours before imaging.

13. Kinetic analysis of tetrazine-mediated protection and GSH-mediated deprotection

Thiol-functionalized polymersomes were incubated with Tz1 or Tz2 at an initial tetrazine concentration of 20 µM at 37°C. Samples were collected at 0, 2, 5, 10, 15, 30, 45, 60, 90, 120, 180, 240, and 360 min. At each time point, the polymersomes were removed by filtration through a 0.22 µm syringe filter, and the concentration of unreacted tetrazine in the filtrate was

determined by reverse-phase HPLC using calibration curves established separately for Tz1 and Tz2. For the kinetic analysis of GSH-mediated deprotection, Tz1- or Tz2-protected polymersomes were first prepared by incubating thiol-functionalized polymersomes (1 mg mL^{-1}, calculated based on PEG–PS) with the corresponding tetrazine (50 µM) for 3 h at 37 °C. Unreacted tetrazine was then removed by repeated centrifugation (14000 rpm, 10 min). The protected polymersomes were subsequently incubated with GSH (5 mM) at 37 °C. Samples were collected at 0, 0.5, 1, 2, 5, 10, 15, 30, 45, 60, 90, 120, and 180 min. The polymersomes were removed immediately after sampling, and the concentrations of the released GSH–Tz1 or GSH–Tz2 conjugates were quantified by HPLC using the corresponding calibration curves.

The protection kinetics were fitted using a one-phase decay model,

$$C(t) = C_{\infty} + (C_0 - C_{\infty}) \times e^{-k_{obs} \times t}$$

where $C(t)$ is the concentration of unreacted tetrazine at time t, C_0 is the initial tetrazine concentration, C_{∞} is the fitted plateau concentration, and k_{obs} is the apparent rate constant.

The deprotection kinetics were fitted using a one-phase association model,

$$P(t) = P_0 + (P_{\infty} - P_0) \times [1 - e^{-k_{obs} \times t}]$$

where $P(t)$ is the concentration of GSH–tetrazine conjugate at time t, P_0 is the initial product concentration, P_{∞} is the fitted plateau concentration, and k_{obs} is the apparent rate constant. Nonlinear regression was performed using GraphPad Prism.

14. Statistical analysis

Statistical analyses were performed using GraphPad Prism. Data in Figures 3b and 3c were analyzed using ordinary one-way analysis of variance (ANOVA), followed by Dunnett's multiple-comparisons test. Data in Figure S5 were analyzed using one-way ANOVA and Dunnett's multiple-comparisons test. Data in Figure S12 were analyzed using one-way ANOVA and Dunnett's multiple-comparisons test. Data in Figure S14 were analyzed using a two-tailed unpaired Student's t-test. Data are presented as mean ± SD unless otherwise stated. $p < 0.05$ (*), $p < 0.01$ (**), and $p < 0.001$ (***).

Supplementary figures

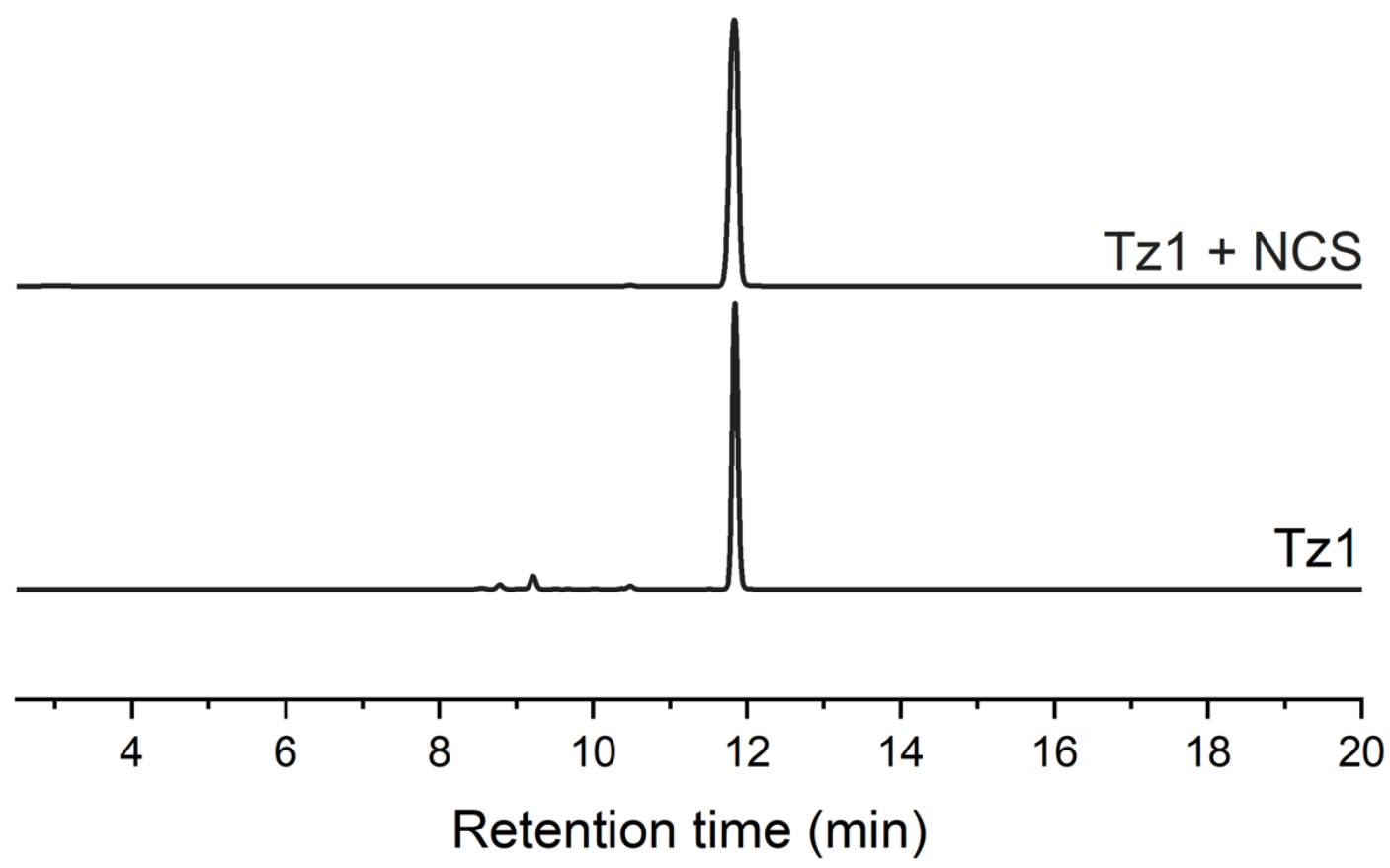

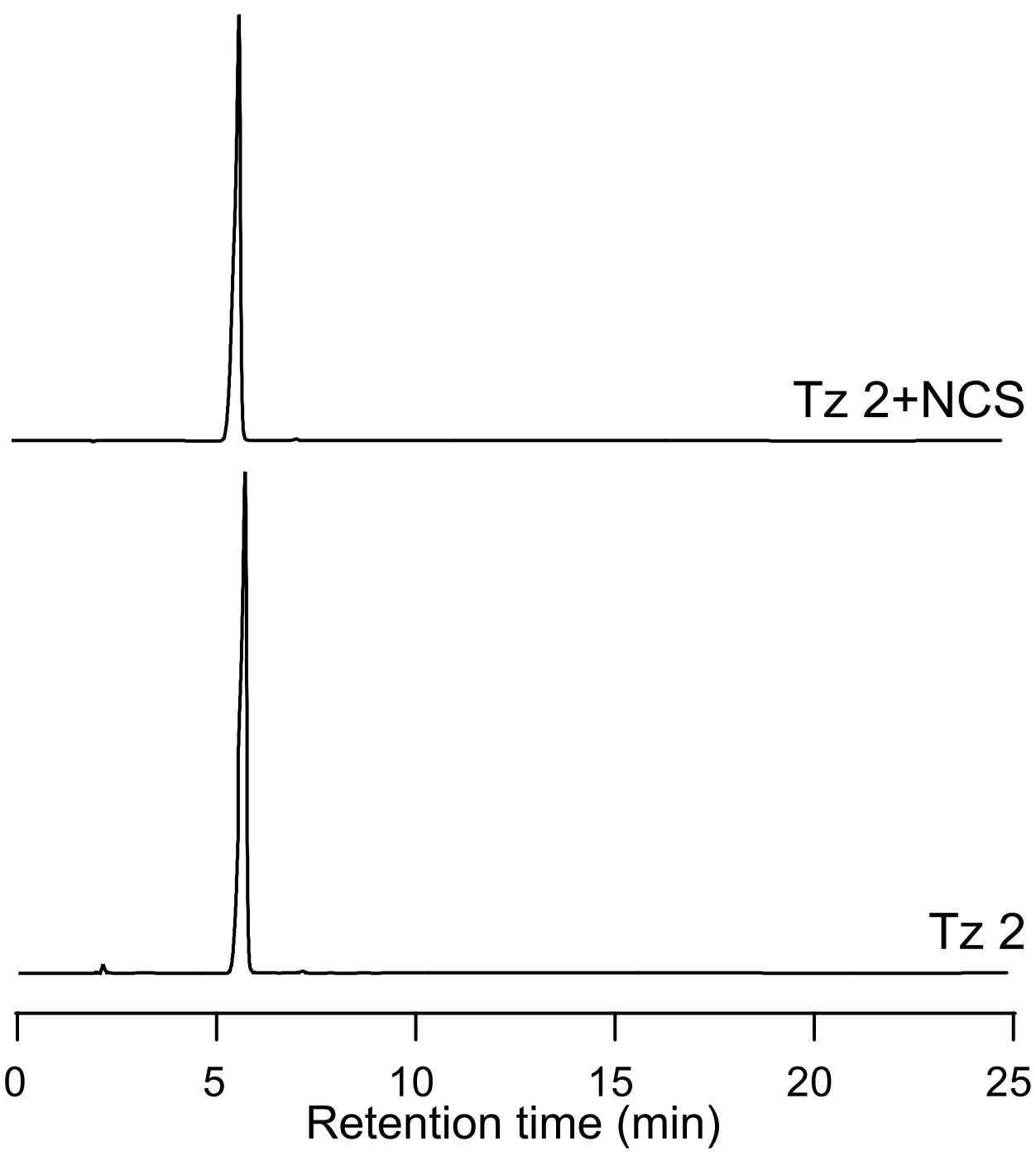

Figure S1. Chemical stability of tetrazines under accelerated oxidative conditions. **Tz1** and **Tz2** at 2 mM were mixed with N-Chlorosuccinimide (NCS, 1 mM) in Milli-Q water for 1 h at 37°C. The preservation of the characteristic tetrazine peaks confirms the stability against a potent electrophilic oxidant**.**

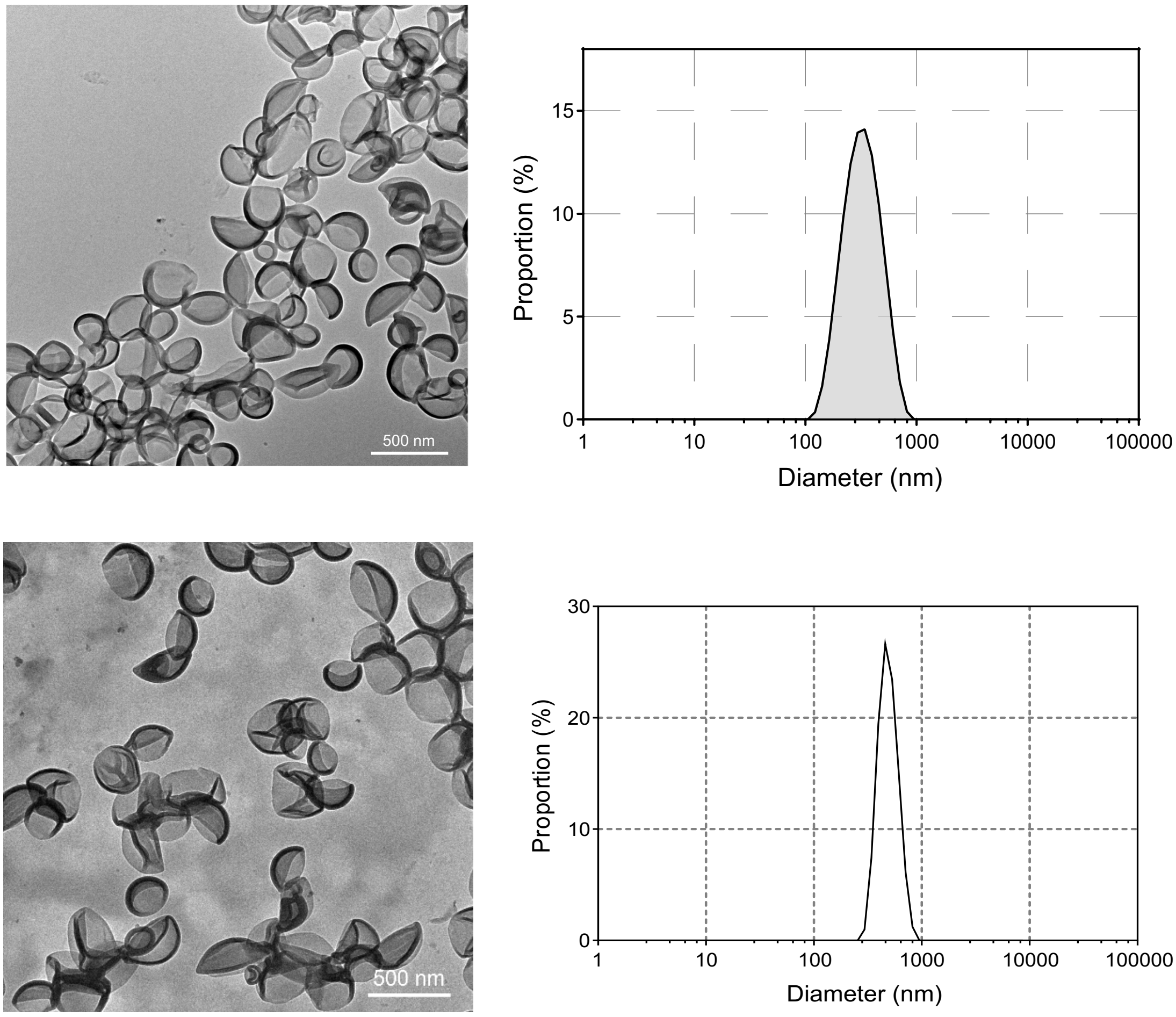

Figure S2. TEM image and size distribution of SH-polymersomes (up) and Nile red-labelled polymersomes (down).

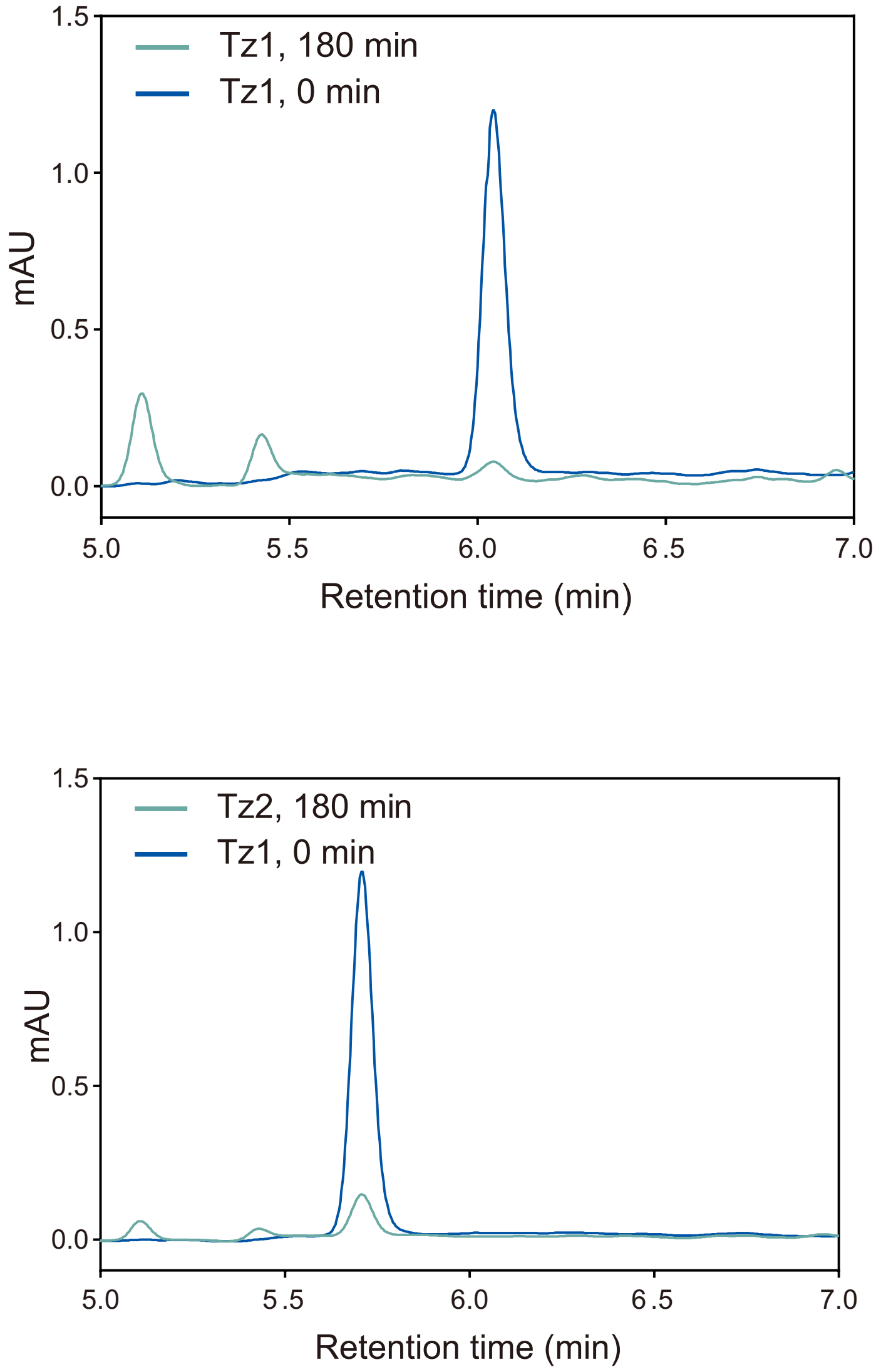

Figure S3. HPLC trace showing **Tz1** (up) and **Tz2** (down) reacting with thiols on the polymersome surface. **Tz1** and **Tz2** were respectively incubated with SH-polymersomes suspension at a final concentration of 0.5 μM for 180 min at pH 7.4, 37°C. The reduction of the peaks (at 6.1 min for **Tz1**; at 5.7 min for **Tz2**) indicates the consumption of tetrazine.

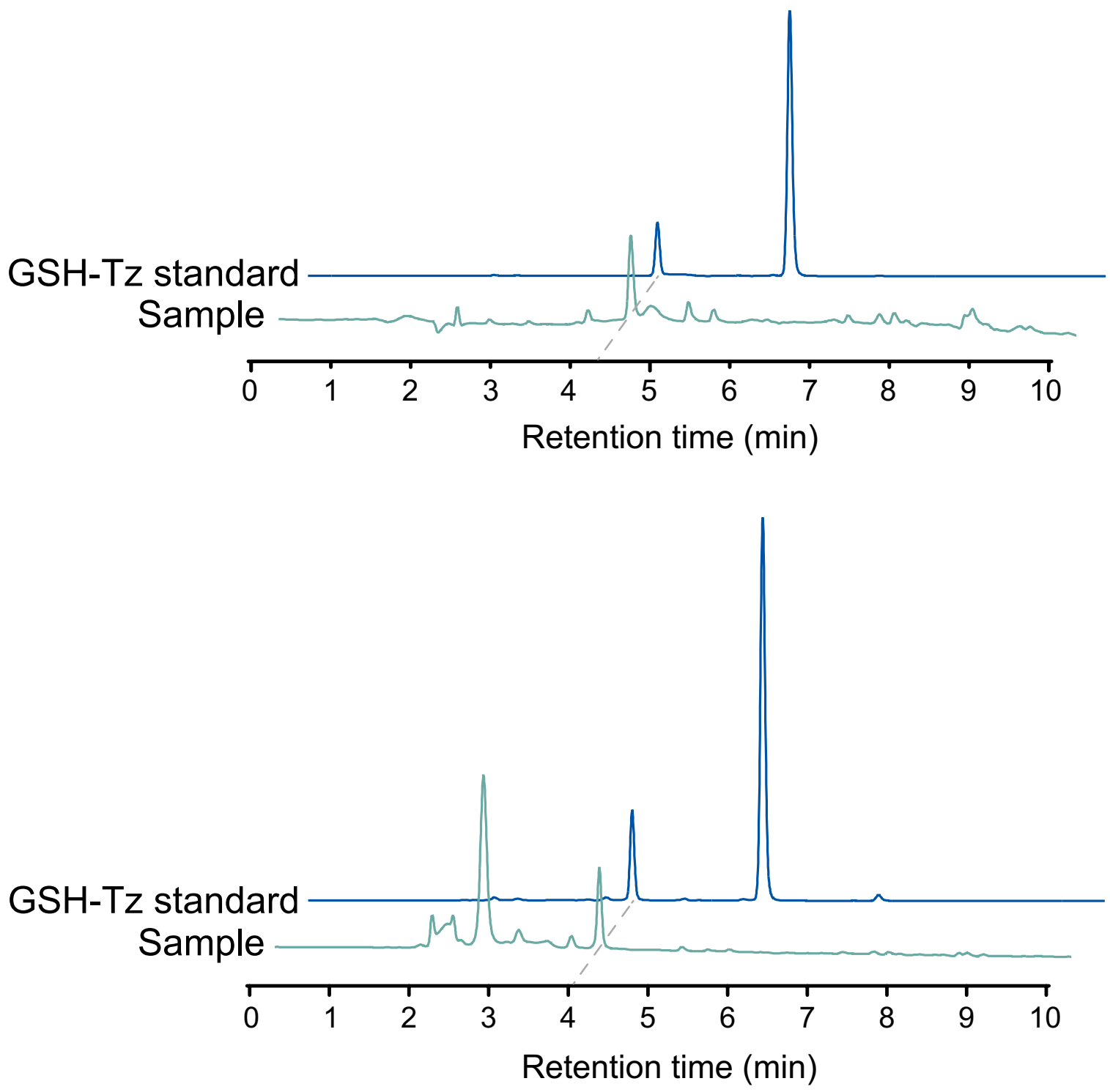

Figure S4. HPLC traces confirming the reversible protection of thiols by tetrazine. Blue lines indicate the retention times of GSH-tetrazine conjugates (GSH-Tz standard, 4.2 min for GSH-**Tz1**, and 4.0 min for GSH-**Tz2**). After the tetrazine protection, polymersomes were incubated with 20 µM of **Tz1** (up) and **Tz2** (bottom) for 180 min at pH 7.4, 37°C. The appearance of peaks in green with the same retention time as the GSH-tetrazine indicates the generation of GSH-tetrazine.

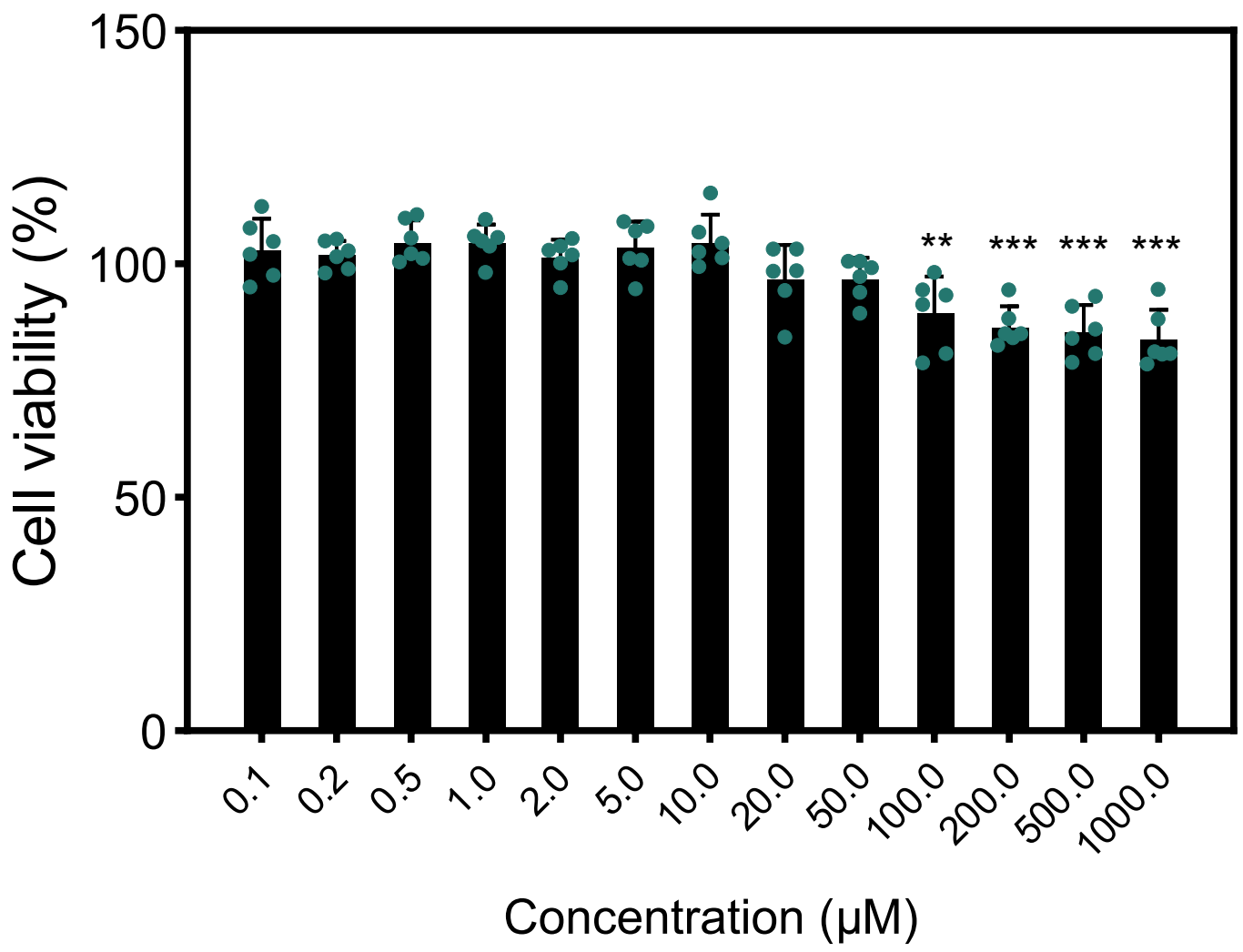

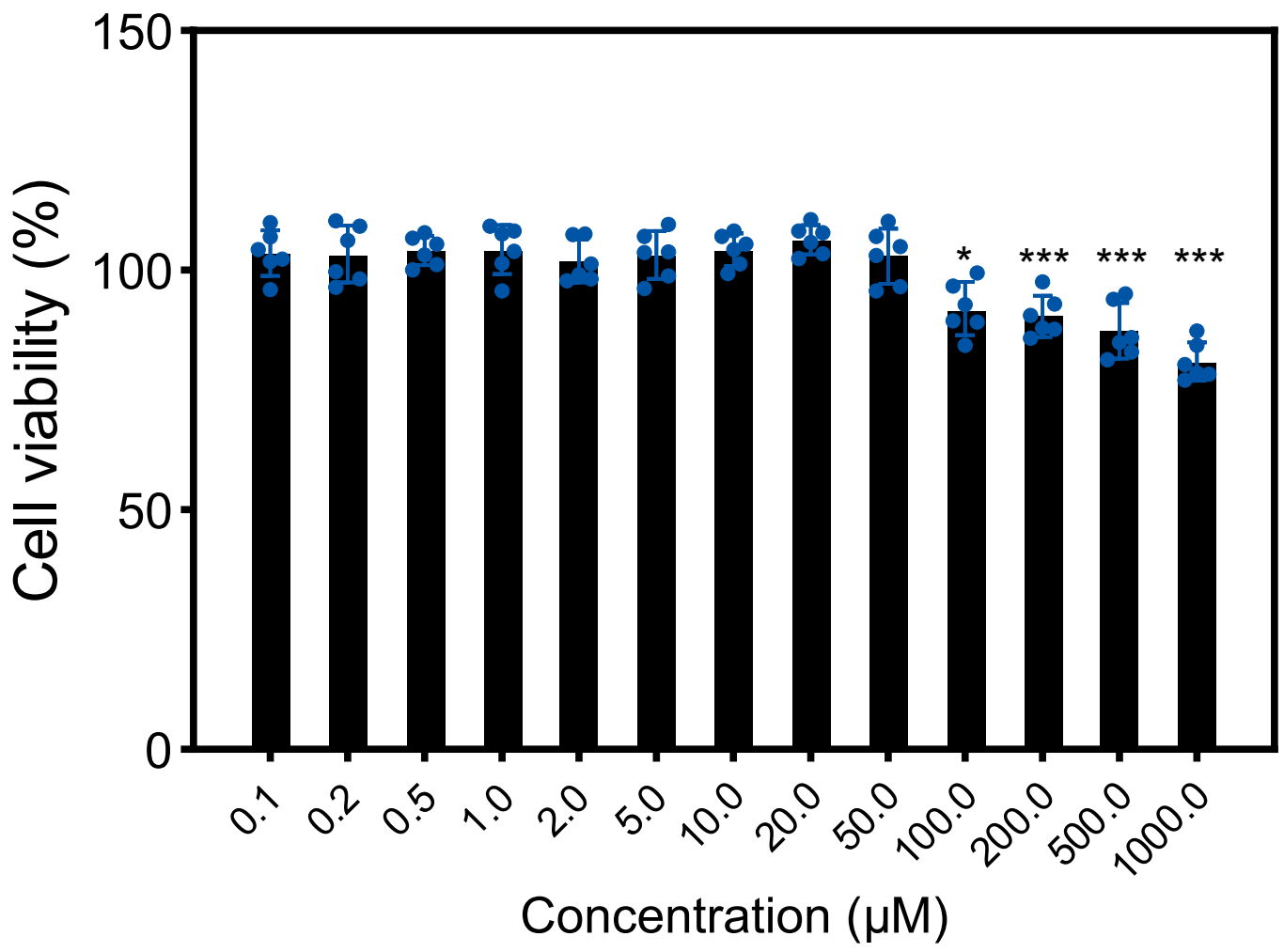

Figure S5. CCK-8-based cell viability assay of Tz1 and Tz2 on NIH-3T3 cells for 72 hours. Data are presented as mean ± SD (n = 6). Statistical significance was assessed using one-way ANOVA followed by Dunnett's multiple-comparisons test against the untreated control. p <0.05 (*), p < 0.01 (**), and p < 0.001 (***).

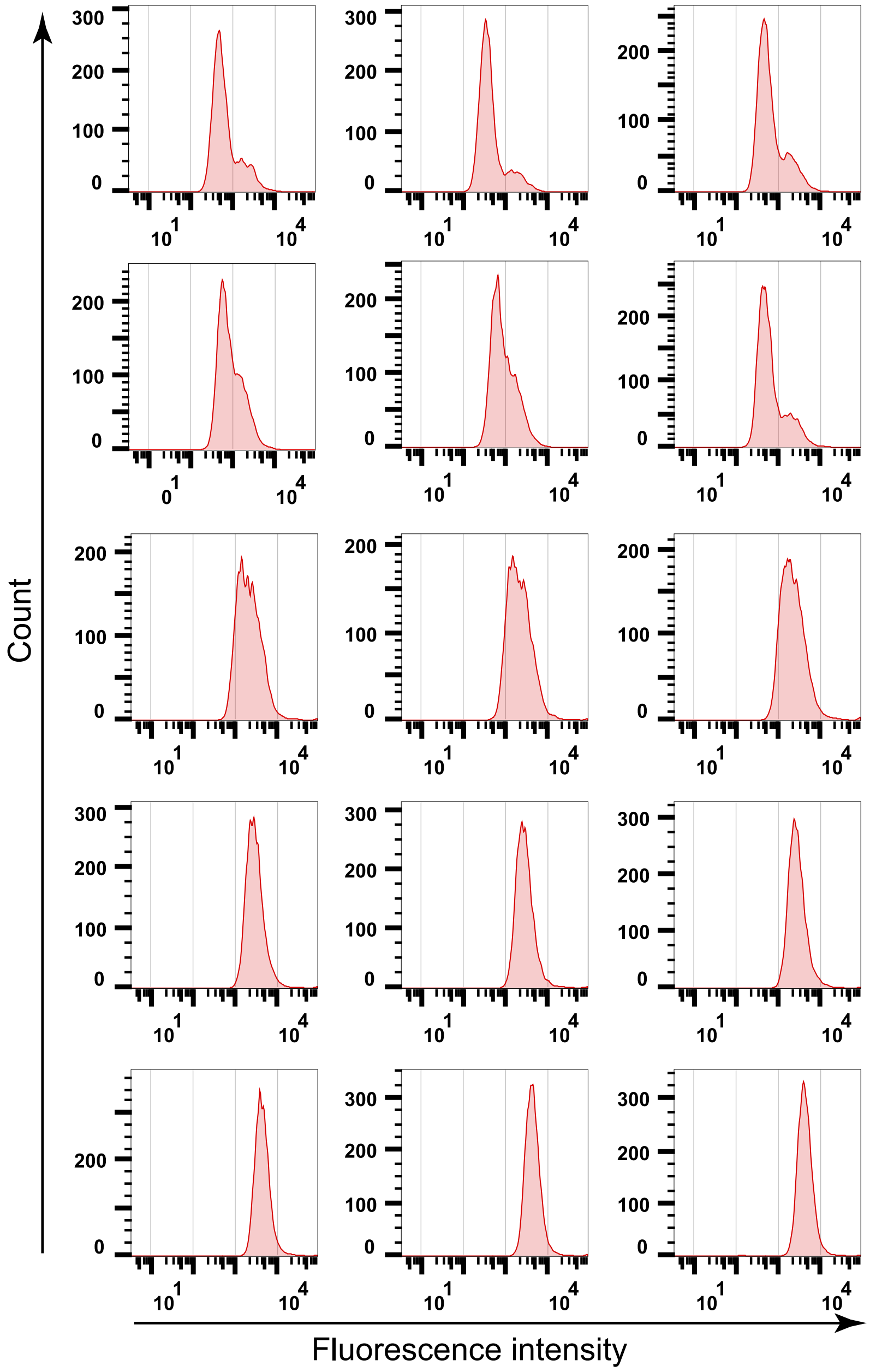

Figure S6. Individual flow cytometry analysis of fluorescence intensity of HeLa cells after being treated with varying concentrations of **Tz1** for 2 h at 37°C. From top to bottom:0, 10 nM, 500 nM, 2 μM, and 100 μM.

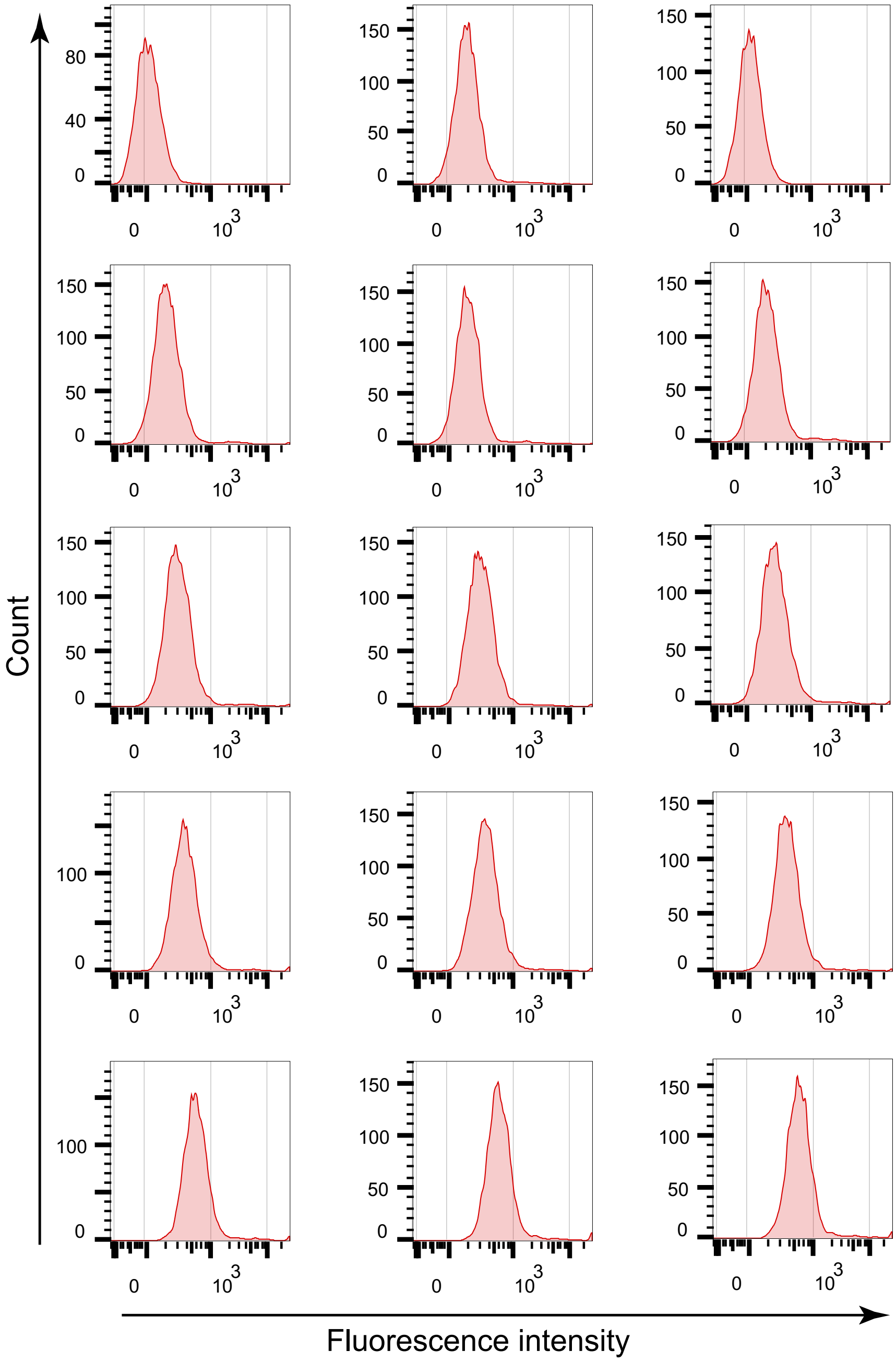

Figure S7. Individual flow cytometry analysis of fluorescence intensity of Jurkat cells after being treated with varying concentrations of **Tz1** for 2 h at 37°C. From top to bottom: 0, 10 nM, 500 nM, 2 μM, and 100 μM.

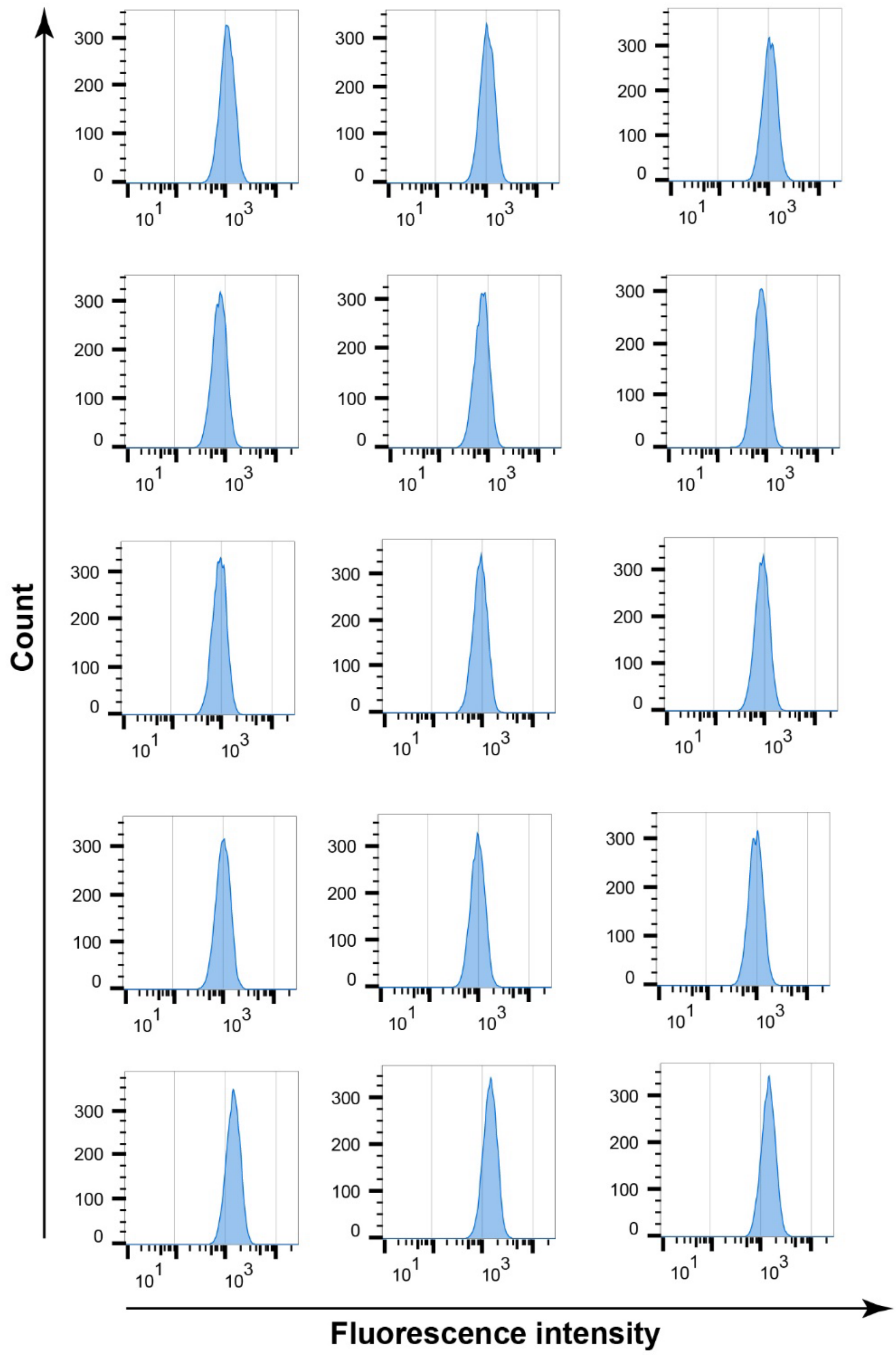

Figure S8. Individual flow cytometry analysis of fluorescence intensity of HeLa cells after being treated with varying concentrations of **Tz2** for 2 h at 37°C. From top to bottom: 0, 10 nM, 500 nM, 2 μM, and 100 μM.

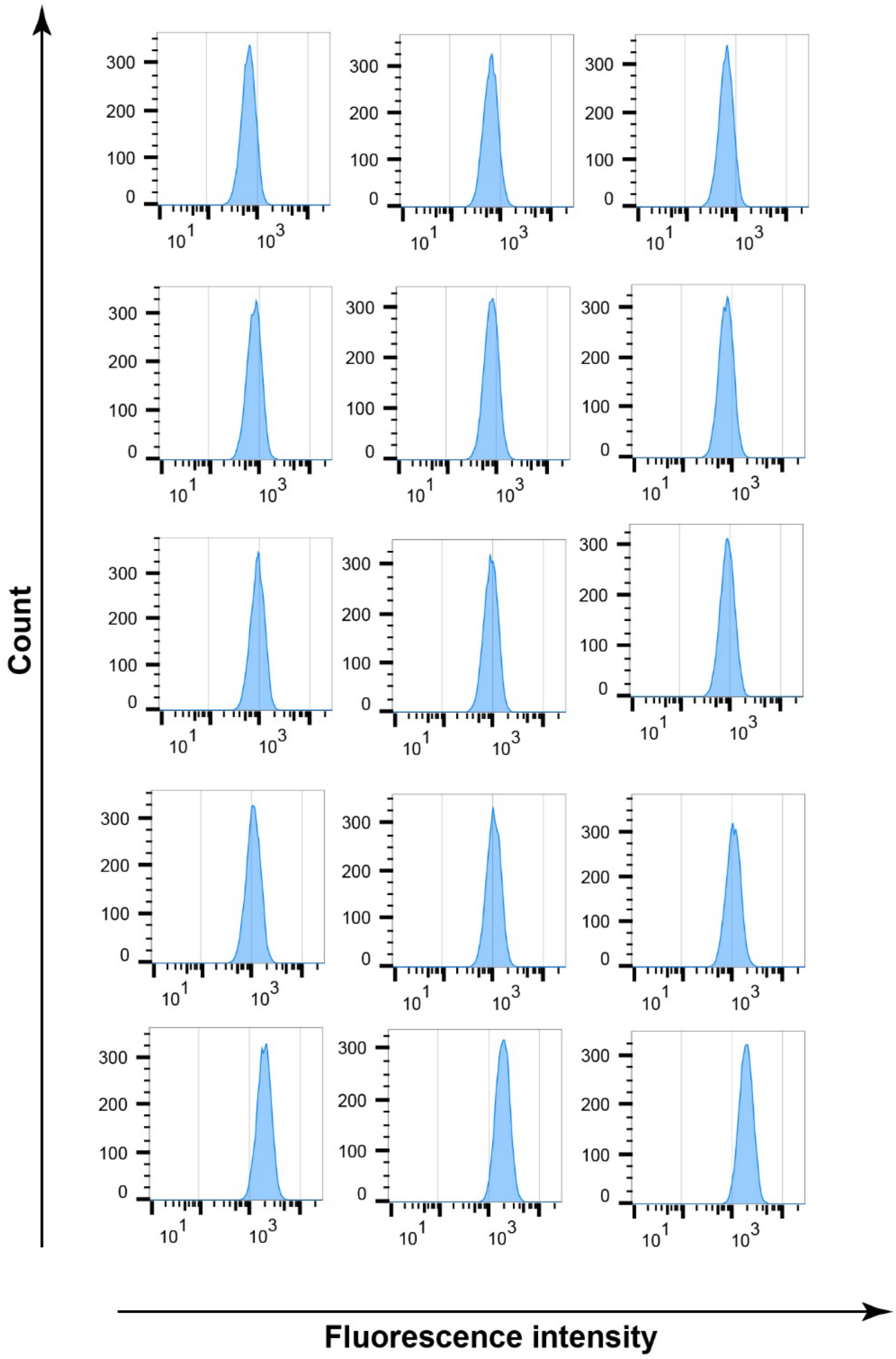

Figure S9. Individual flow cytometry analysis of fluorescence intensity of Jurkat cells (Figure 3) after being treated with varying concentrations of **Tz2** for 2 h at 37°C. From top to bottom: 0, 10 nM, 500 nM, 2 µM, and 100 µM.

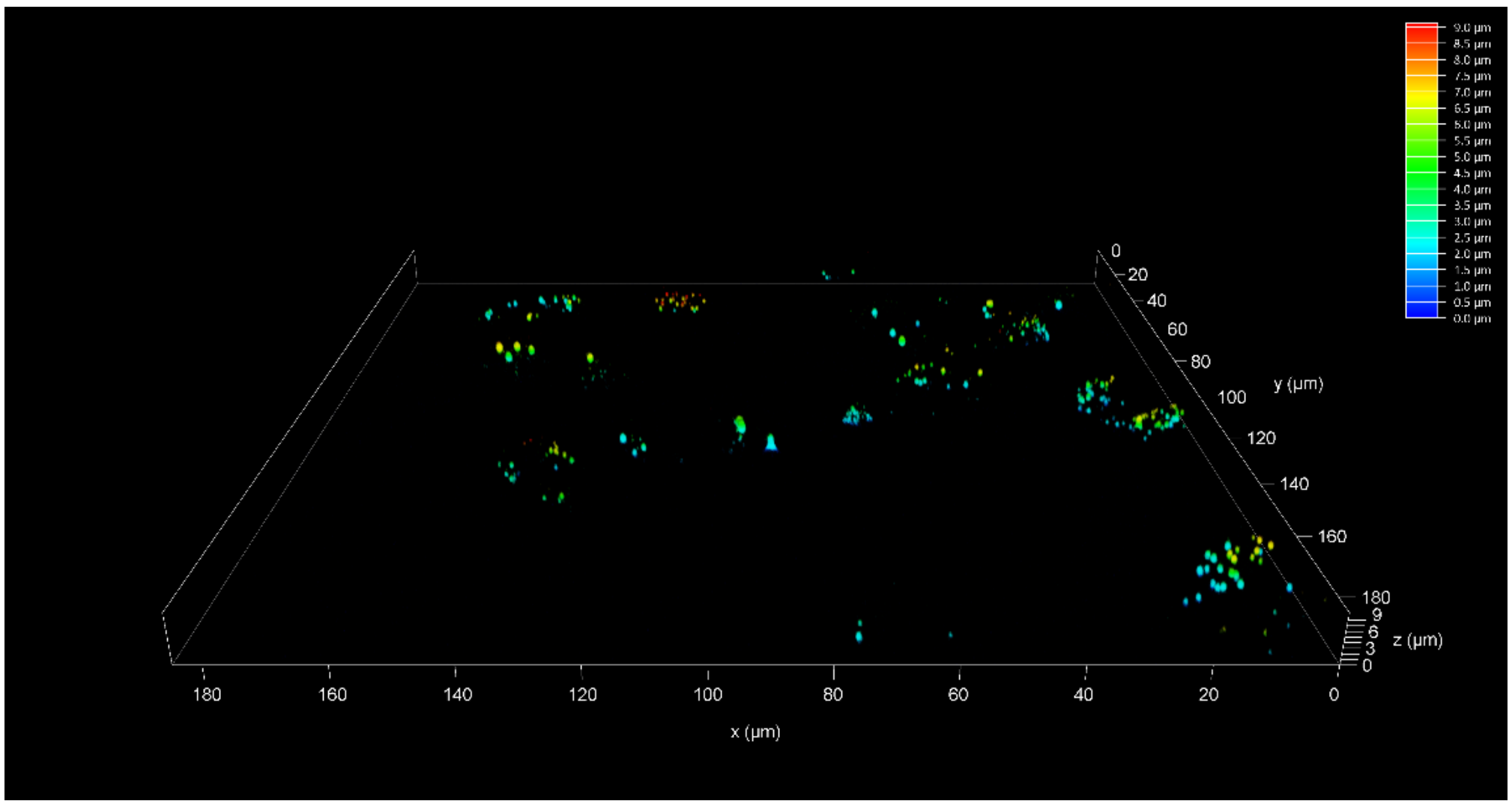
x (µm)
y (µm)
z (µm)

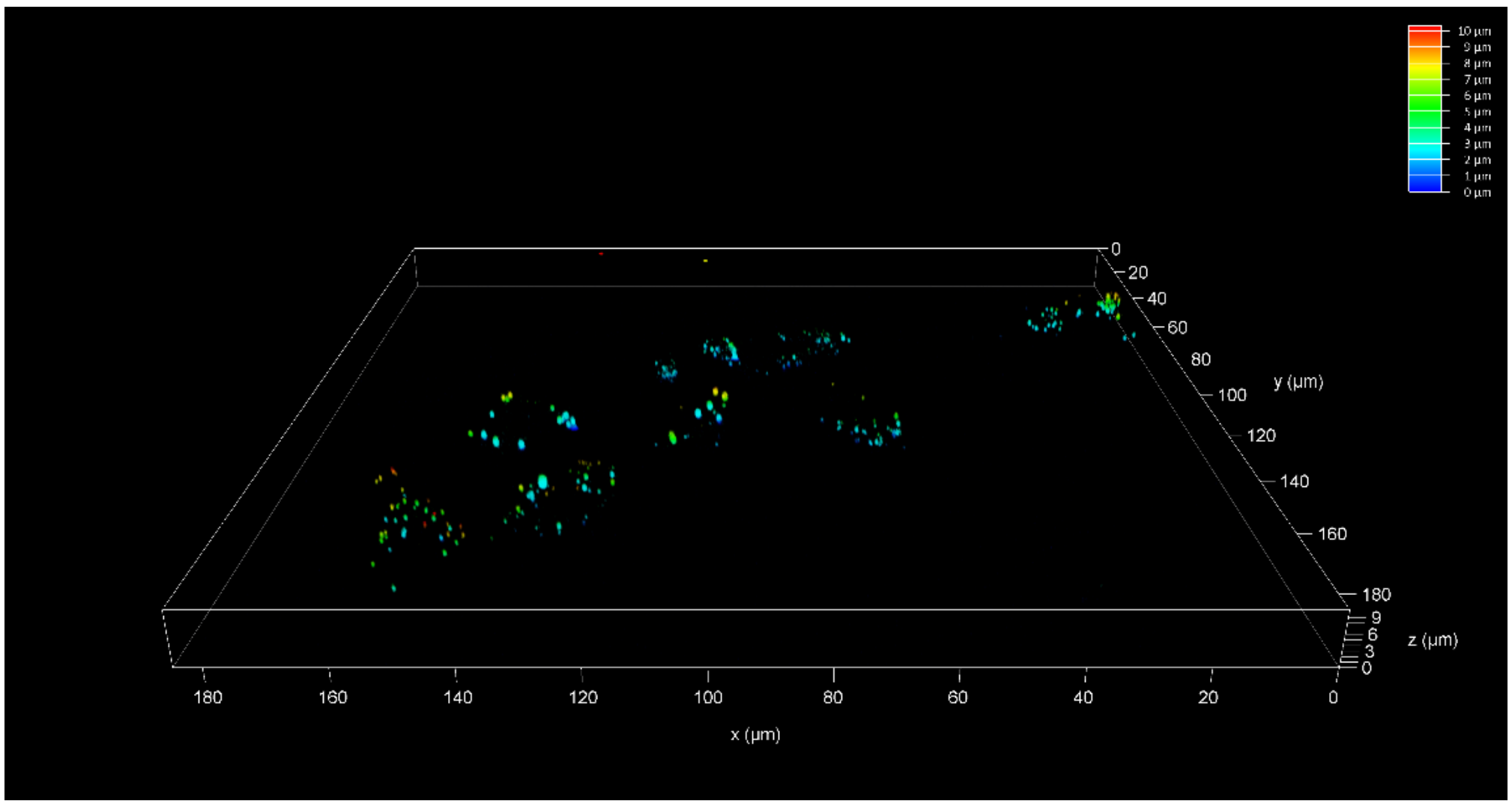
x (µm)
y (µm)
z (µm)

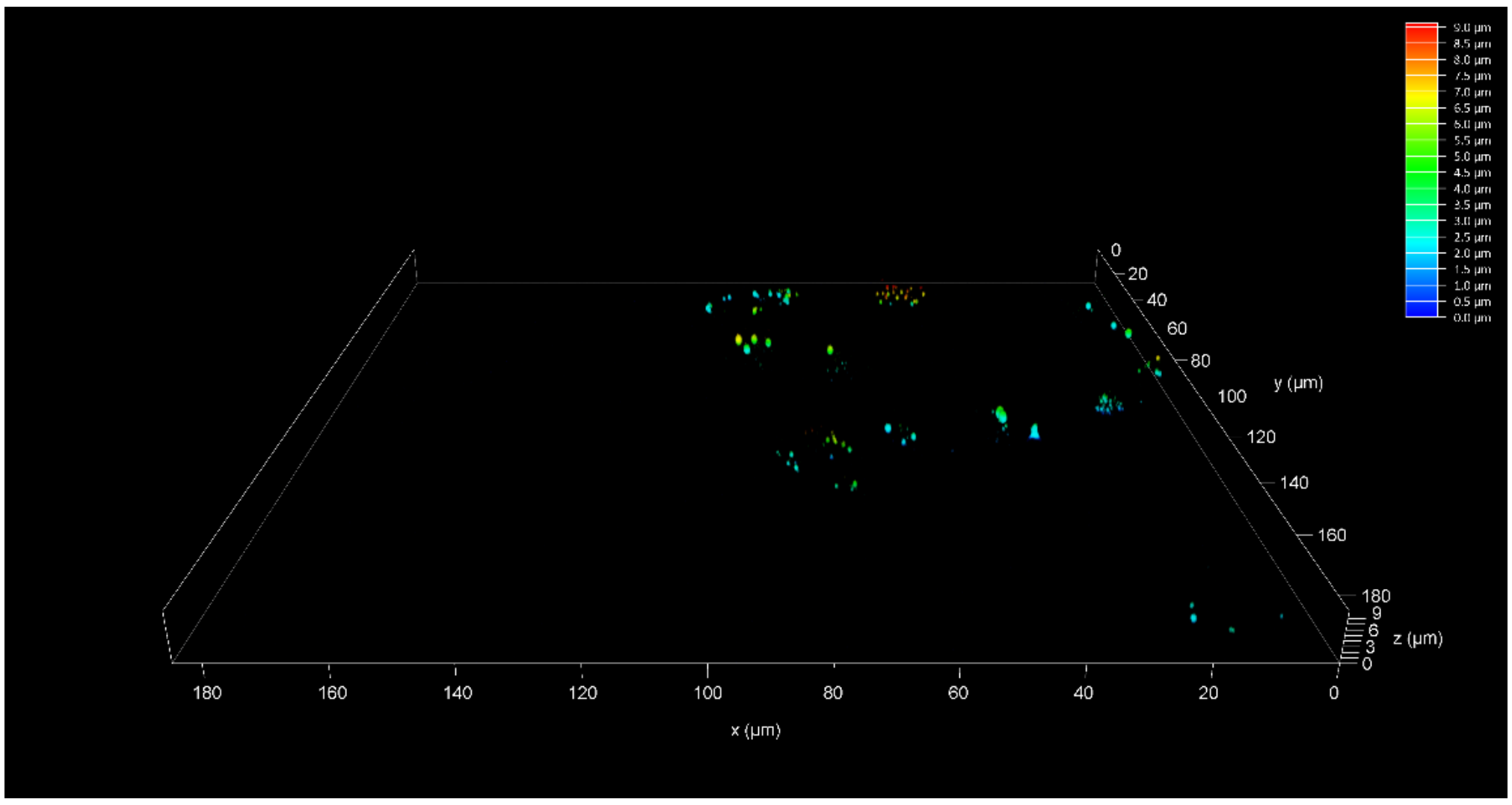
x (µm)
y (µm)
z (µm)

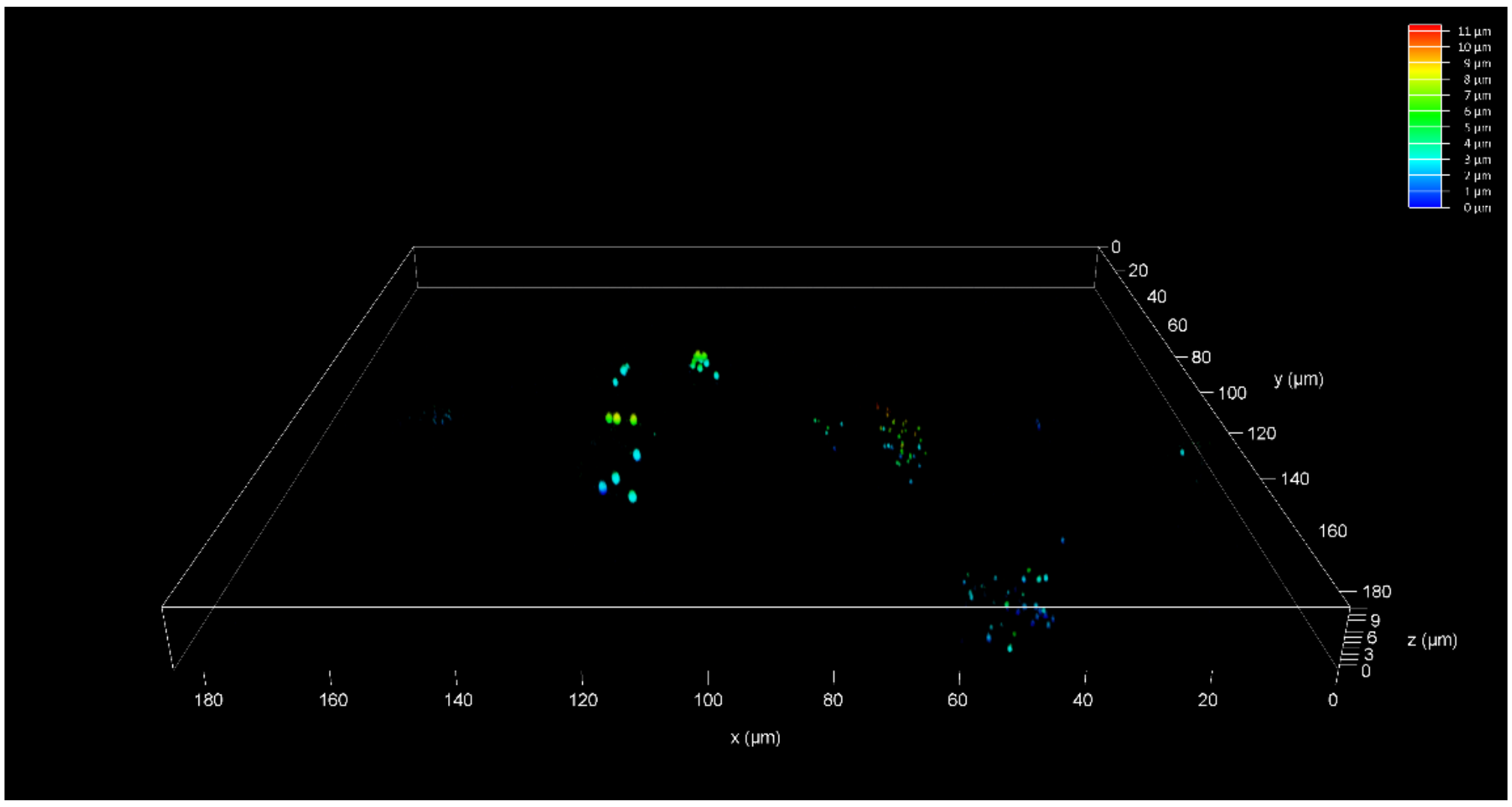

Figure S10. 3D reconstruction of fluorescence images showing cellular uptake by HeLa cells. Cells were pretreated with TCEP (0.5 mM, 30 min at 37℃) and subjected to the following conditions (from top to bottom): (a) PBS (control); (b) **Tz1** (500 nM, 4 h), NaIO4 (2 mM, 1 h), and GSH (2 mM, 2 h); (c) **Tz1** (500 nM, 4 h); and (d) NaIO4 (2 mM, 1 h). Polymersomes were incubated with cells at 300 µg/mL (polymer mass concentration) for 3 hours before imaging.

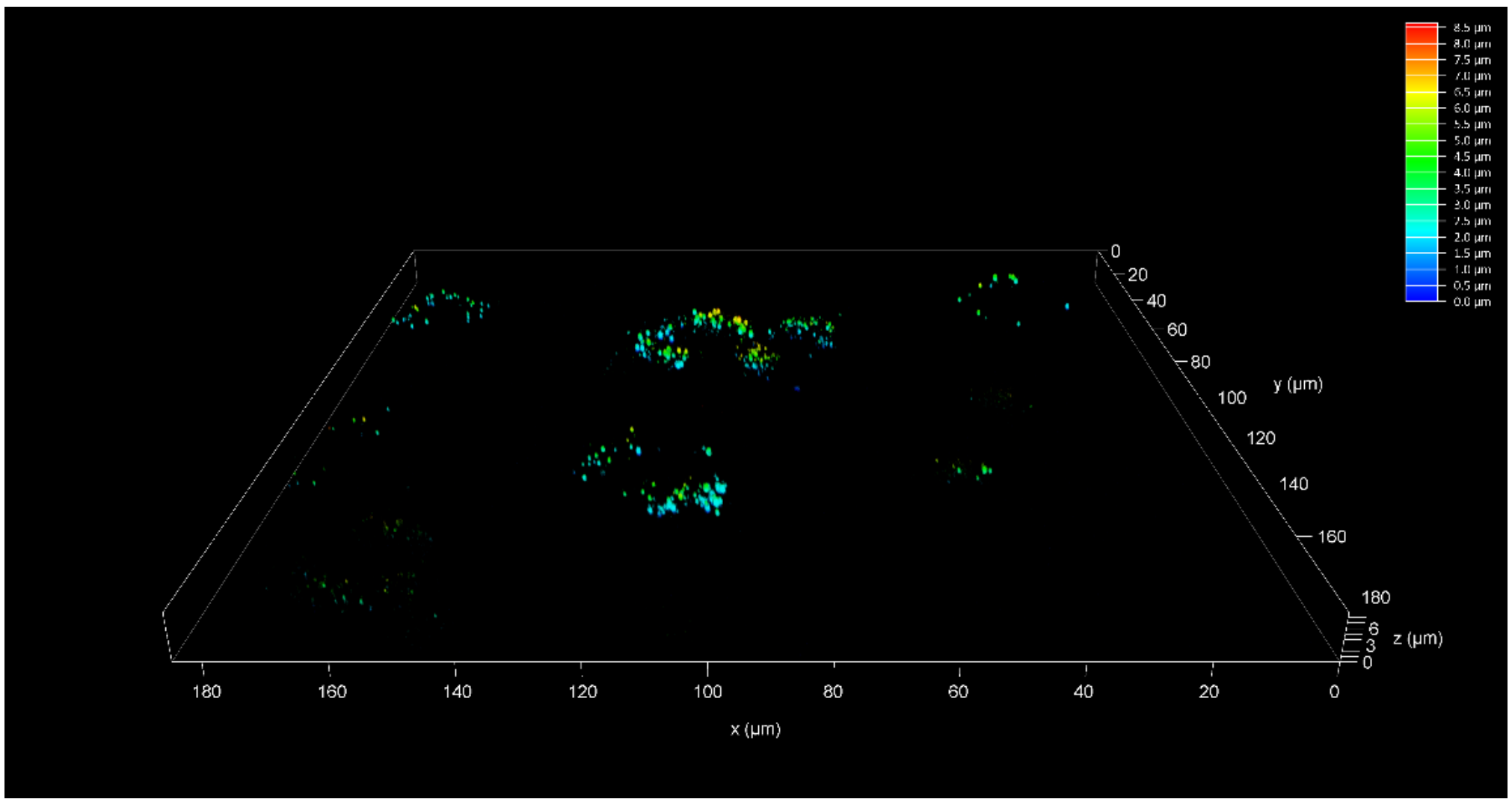

x (µm)
y (µm)
z (µm)

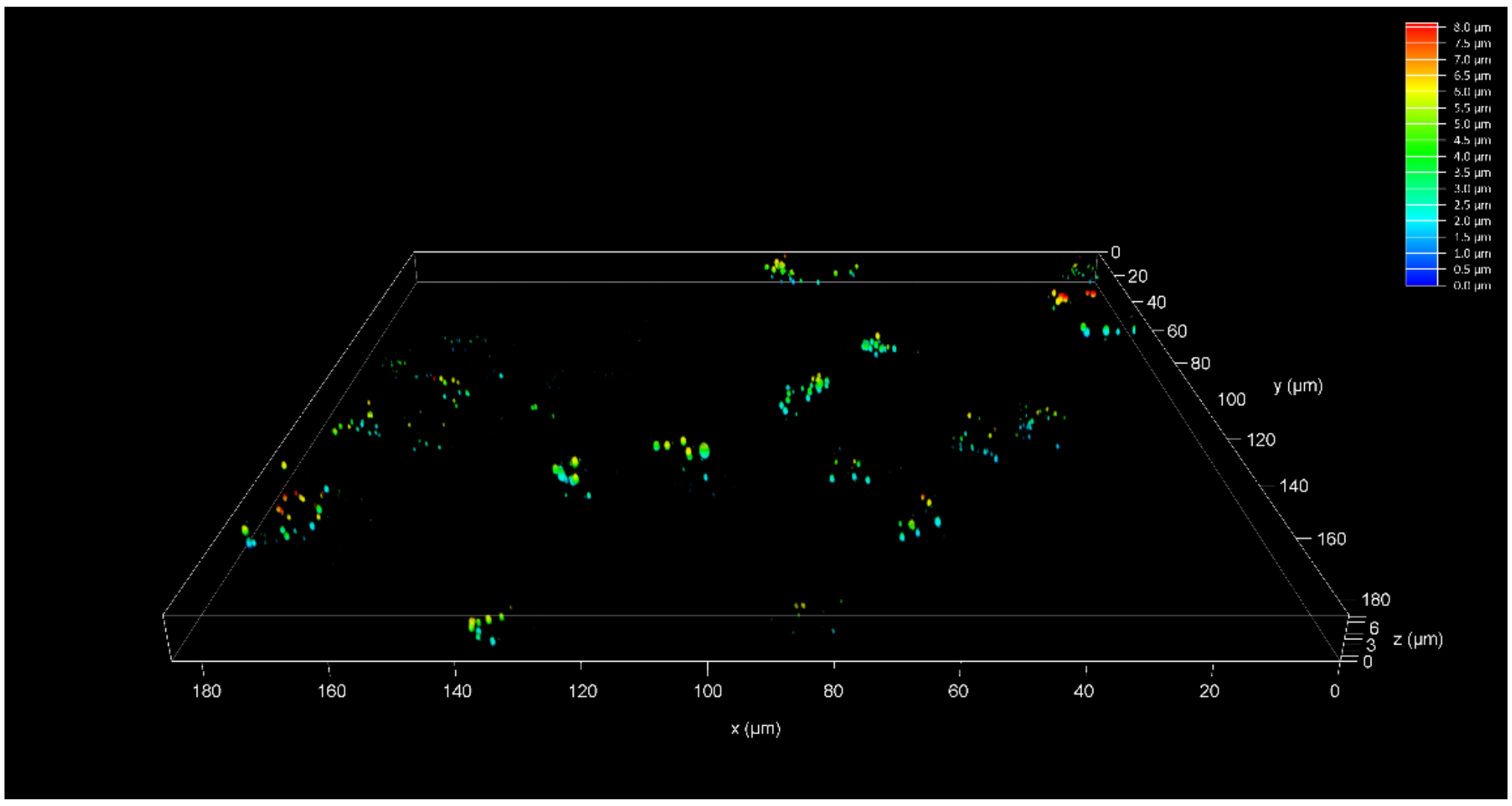

x (µm)
y (µm)
z (µm)

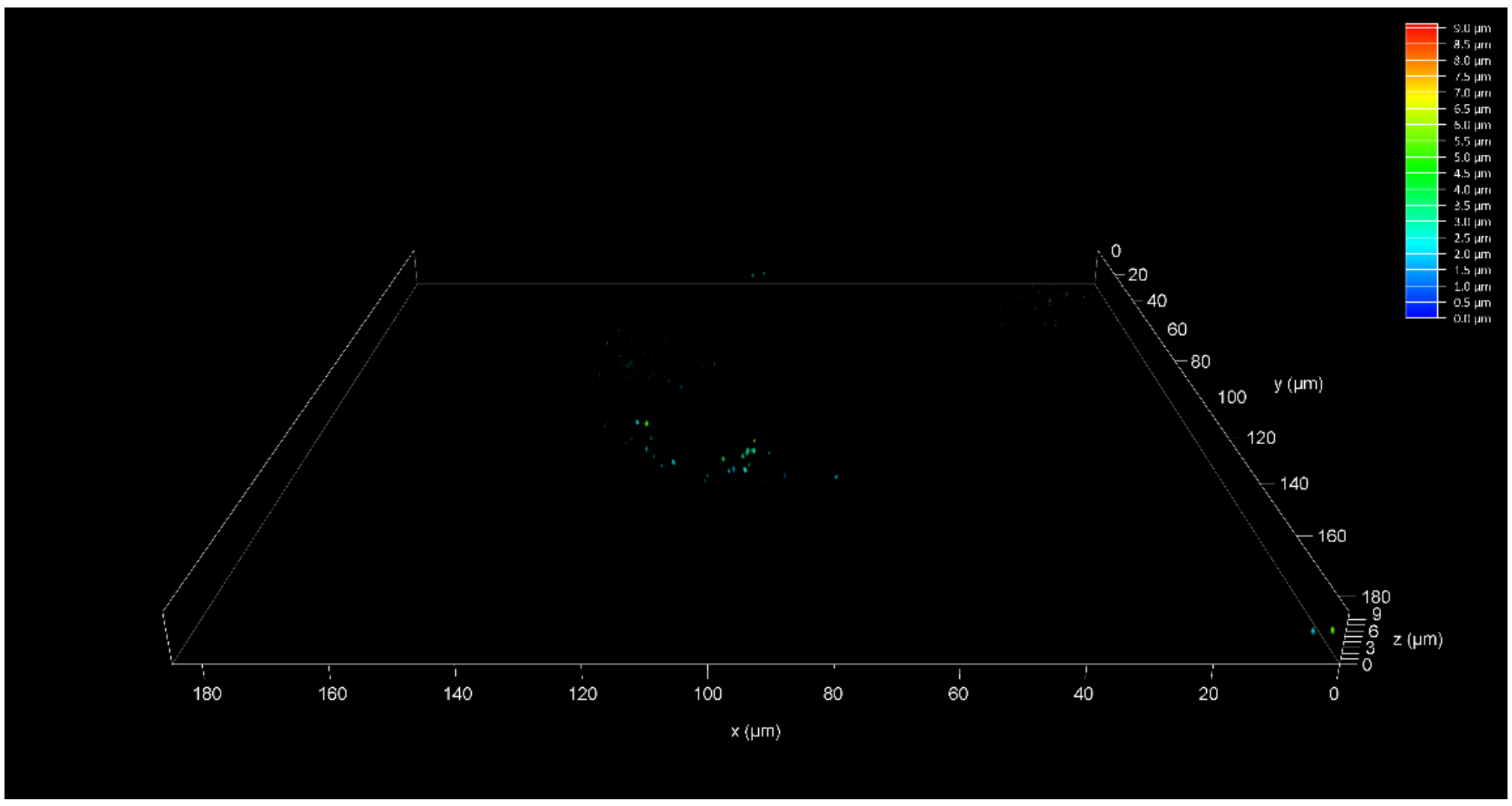

x (µm)
y (µm)
z (µm)

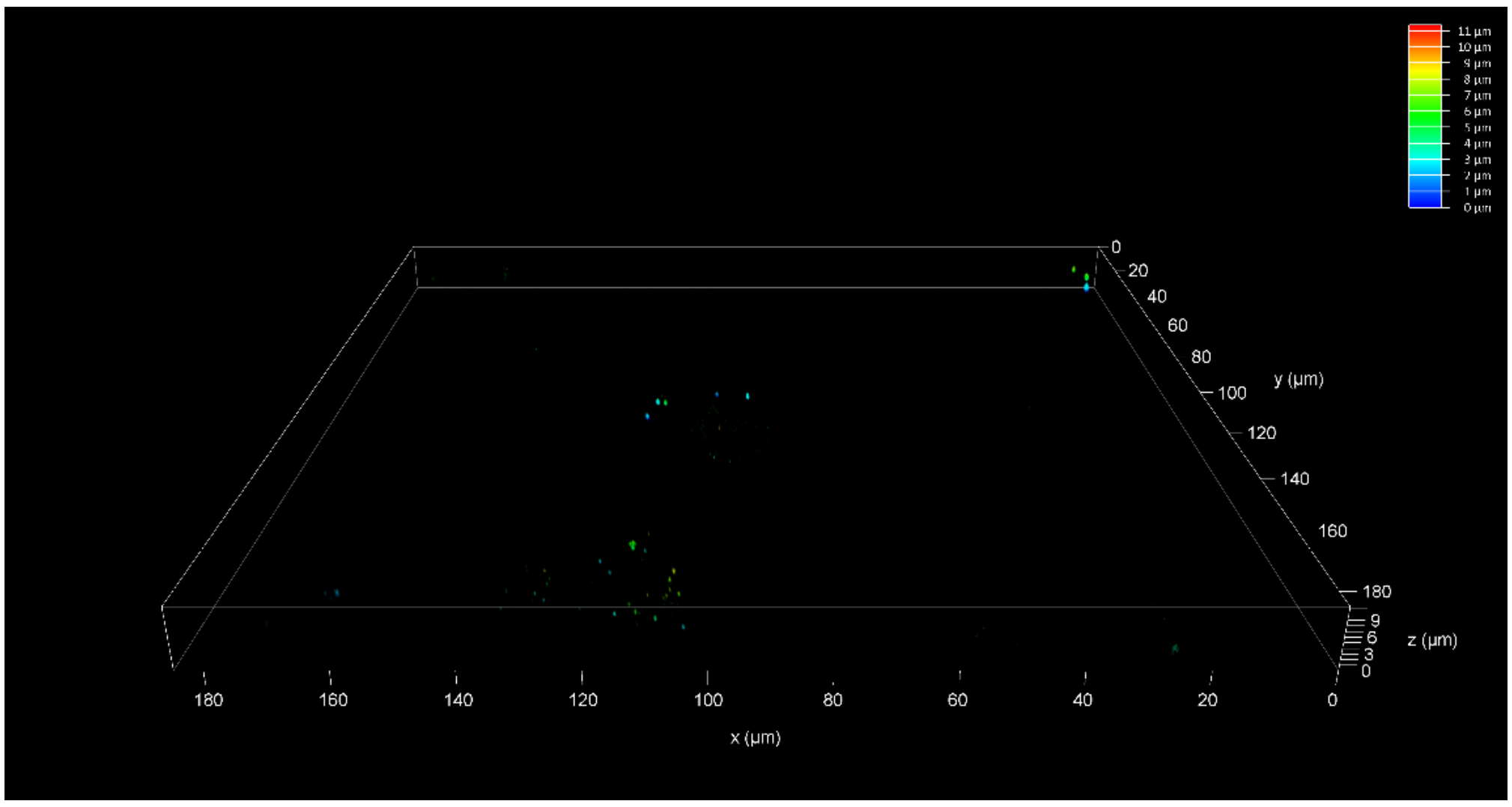

Figure S11. 3D reconstruction of fluorescence images showing cellular uptake by NIH-3T3 cells. Cells were pretreated with TCEP (0.5 mM, 30 min at 37℃) and subjected to the following conditions (from top to bottom): (a) PBS (control); (b) **Tz1** (500 nM, 4 h), NaIO4 (2 mM, 1 h), and GSH (2 mM, 2 h); (c) **Tz1** (500 nM, 4 h); and (d) NaIO4 (2 mM, 1 h). Polymersomes were incubated with cells at 300 µg/mL (polymer mass concentration) for 3 hours before imaging.

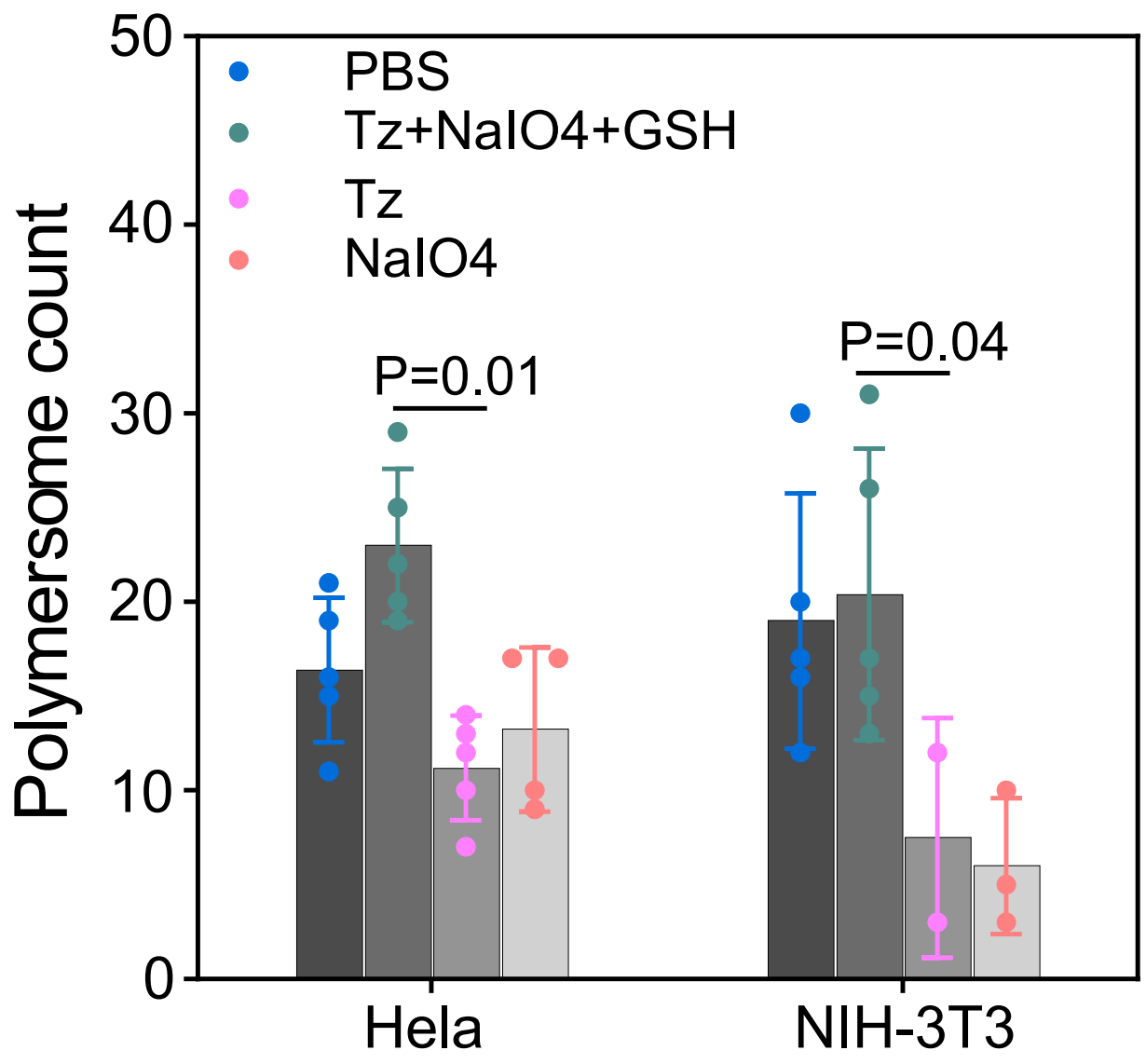

Figure S12. Quantification of polymersome-associated fluorescence in HeLa and NIH-3T3 cells shown in Figure 4c. Maximum-intensity projections were generated from the confocal z-stack images, and Nile Red-positive fluorescent puncta per cell were quantified using ImageJ (n =5). Statistical significance was assessed using ordinary one-way ANOVA followed by Dunnett's multiple-comparisons test.

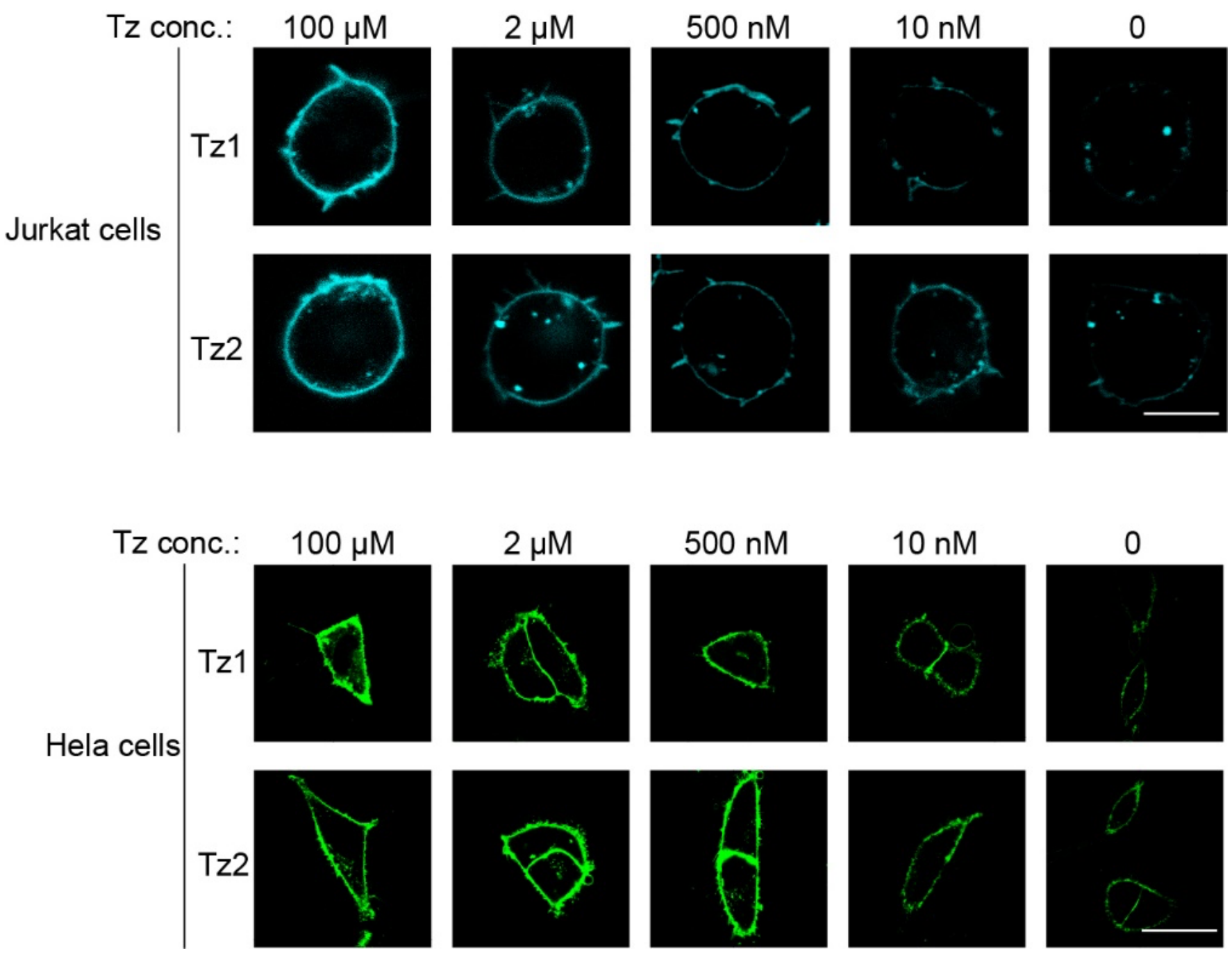

Figure S13. Fluorescence images of Jurkat cells and HeLa cells treated with varying concentrations of tetrazines, as supplementary images for Figure 3b and 3c. Scale bar = 10 μm.

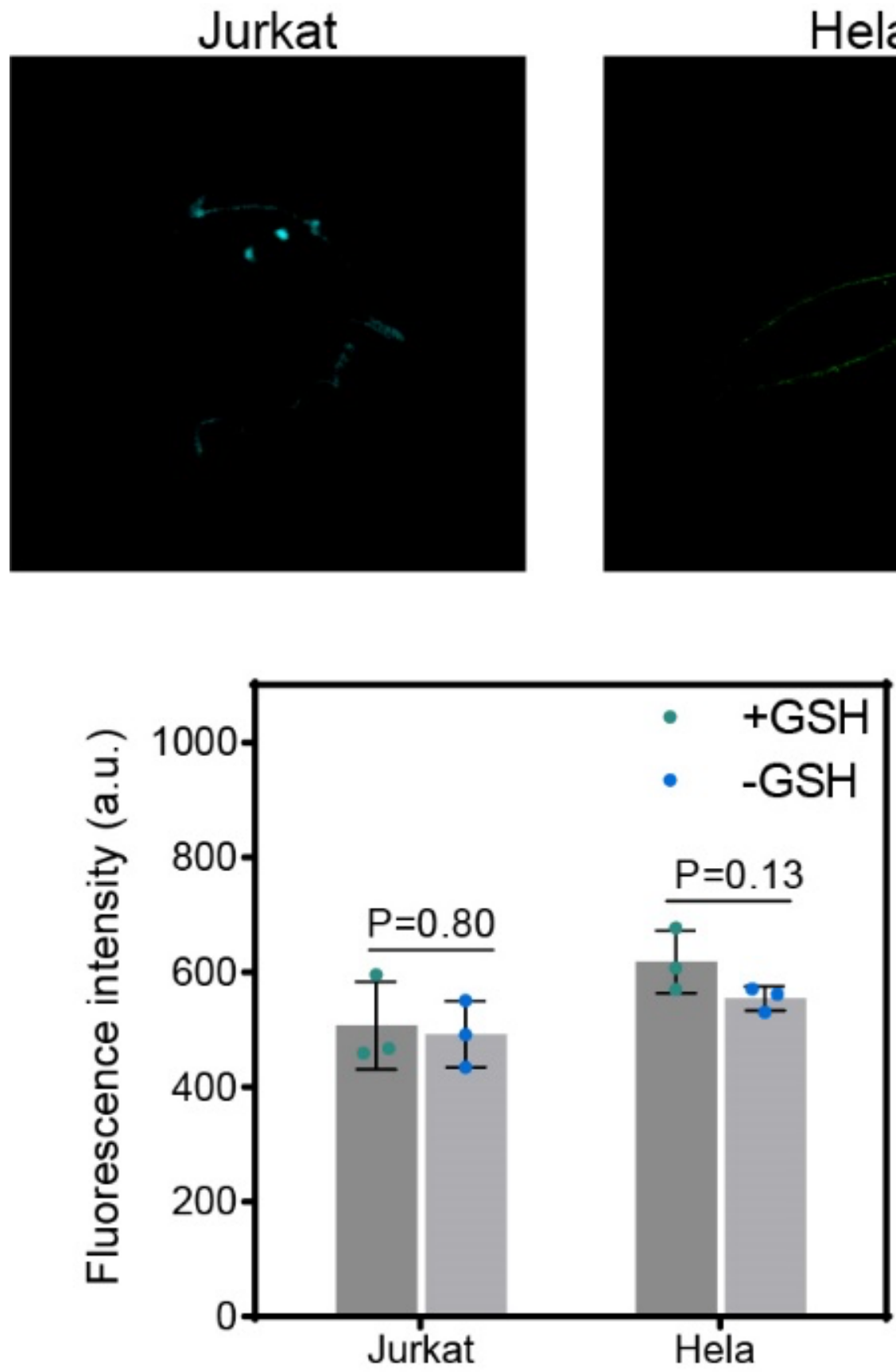

Figure S14. Top: Confocal fluorescence images of Jurkat and HeLa cells treated with either 0 nM **Tz2** followed by GSH or 100 μM **Tz2** without GSH, after $NaIO_4$ oxidation and 5-FM labelling. Scale bar = 10 μm. Bottom: Quantification of fluorescence intensity. The similarly low fluorescence in both groups indicates that residual unmasked thiols escaping $NaIO_4$ oxidation contributed only minimally to the final 5-FM signal. Data are presented as mean ± SD. Statistical significance was assessed using a two-tailed unpaired Student's *t*-test.

Table S1. Time-dependent consumption of **Tz1** and **Tz2** during protection of thiol-functionalized polymersomes.

Time (min)	Tz1 concentration (μM)	Tz2 concentration (μM)
0	20.00	20.00
2	18.8	18.55
5	17.46	17.9
10	15.56	16.3
15	14.46	15.74
30	11.52	12.69
45	10.3	11.75
60	8.15	8.12
90	7.9	7.89
120	6.53	6.55
180	5.28	5.45
240	5.07	5.8
360	5.03	5.3

Table S2. Kinetic parameters for tetrazine-mediated protection of thiol-functionalized polymersomes.

Parameter	Tz1	Tz2
k_{obs} (min^{-1})	0.02542	0.02130
95% CI of k_{obs} (min^{-1})	0.02052, 0.03153	0.01760, 0.02571
$t_{1/2}$ (min)	27.26	32.55
R^2	0.9908	0.9914

Table S3. Time-dependent formation of **GSH–Tz1** and **GSH–Tz2** during GSH-mediated deprotection of tetrazine-protected thiol-functionalized polymersomes.

Time (min)	GSH-Tz1 concentration (μM)	GSH-Tz2 concentration (μM)
0	0	0
0.5	0.79	0.39
1	1.28	0.78
2	1.93	1.51
5	2.96	3.69
10	4.8	6.22
15	6.55	8.76
30	10.03	12.05
45	11.88	14.08
60	12.87	14.71
90	13.68	15.25
120	13.91	15.48

180	14.05	15.99

Table S4. Kinetic parameters for GSH-mediated deprotection of tetrazine-protected polymersomes.

Parameter	Tz1	Tz2
k_{obs} (min^{-1})	0.04014	0.05181
95% CI of k_{obs} (min^{-1})	0.03695, 0.04359	0.04845, 0.05539
$t_{1/2}$ (min)	17.27	13.38
R^2	0.9987	0.9991

Synthesis

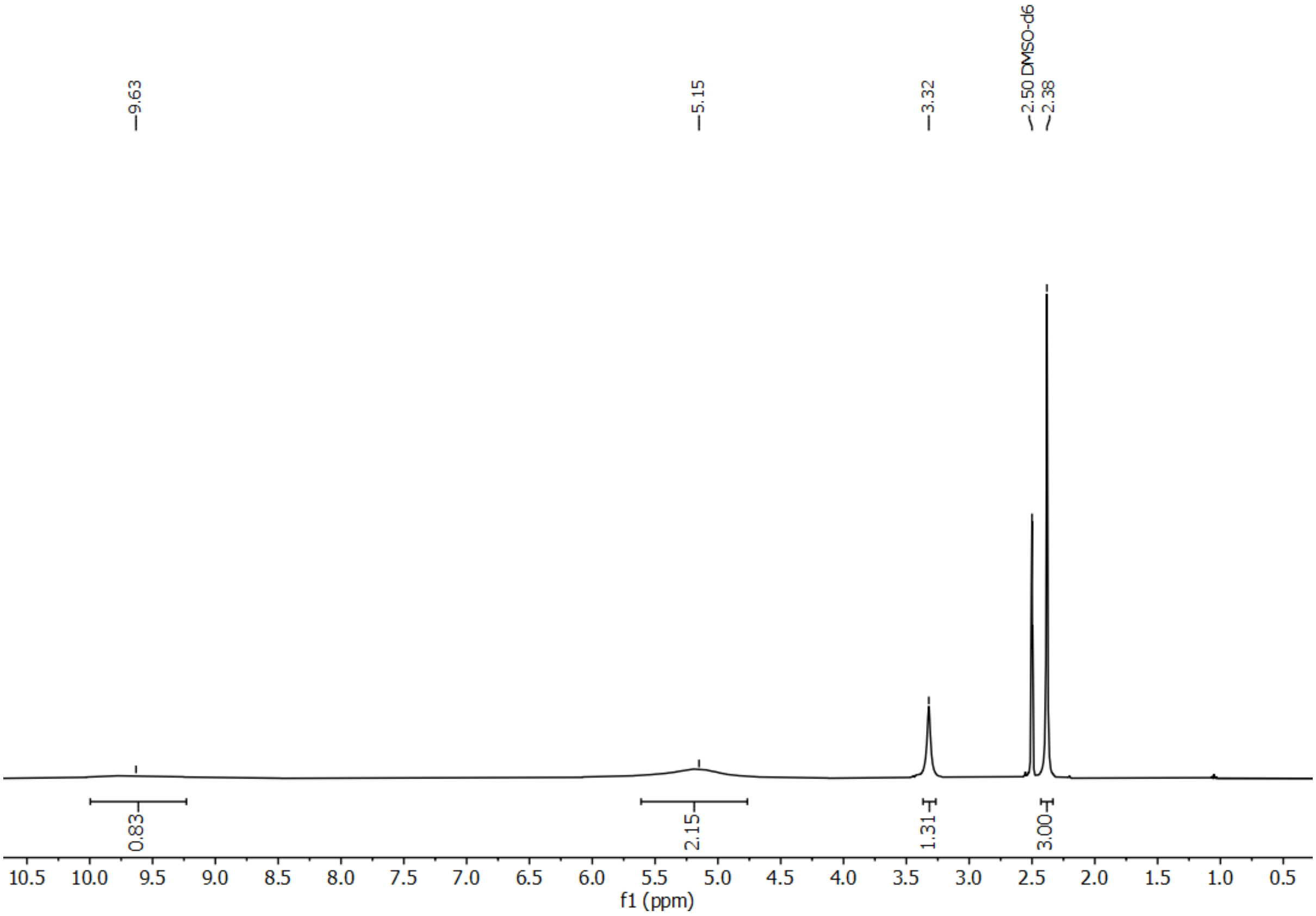

^{1}H-NMR of Methyl thiocarbohydrazide hydrogen iodide salt (**1**).

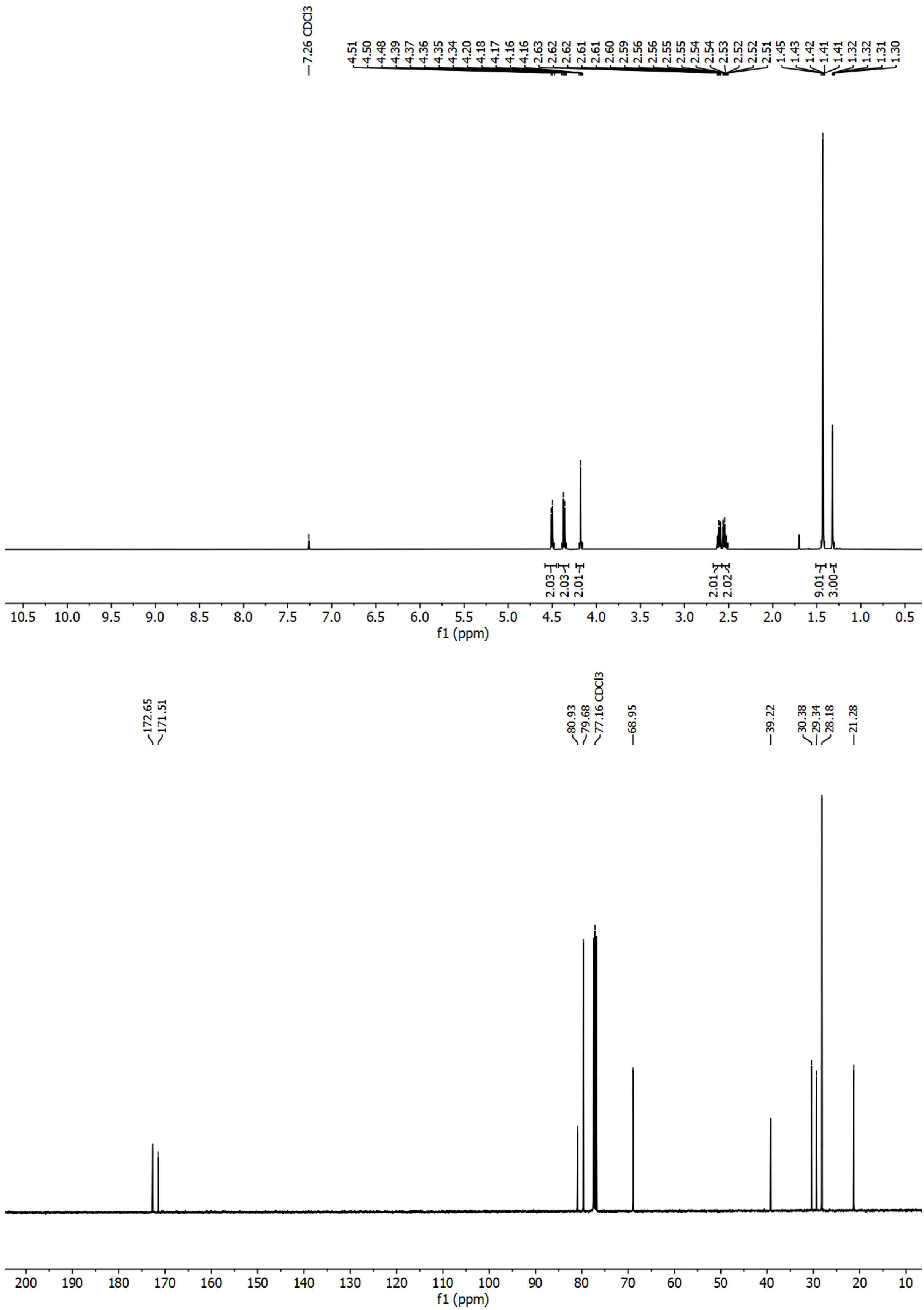

^{1}H-NMR and ^{13}C-NMR of *tert*-Butyl [(3-methyloxetan-3-yl)methyl]succinate (**2**).

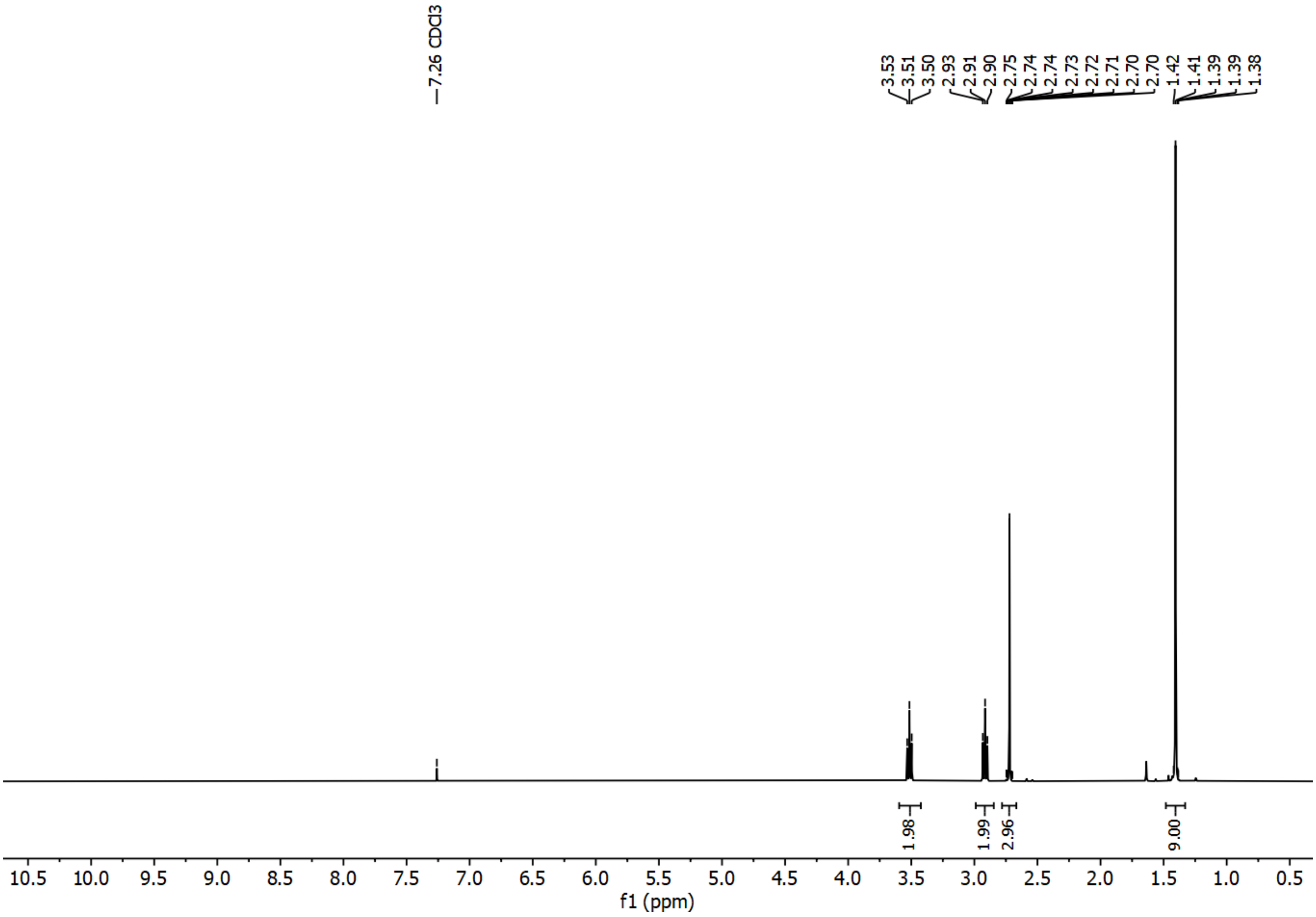

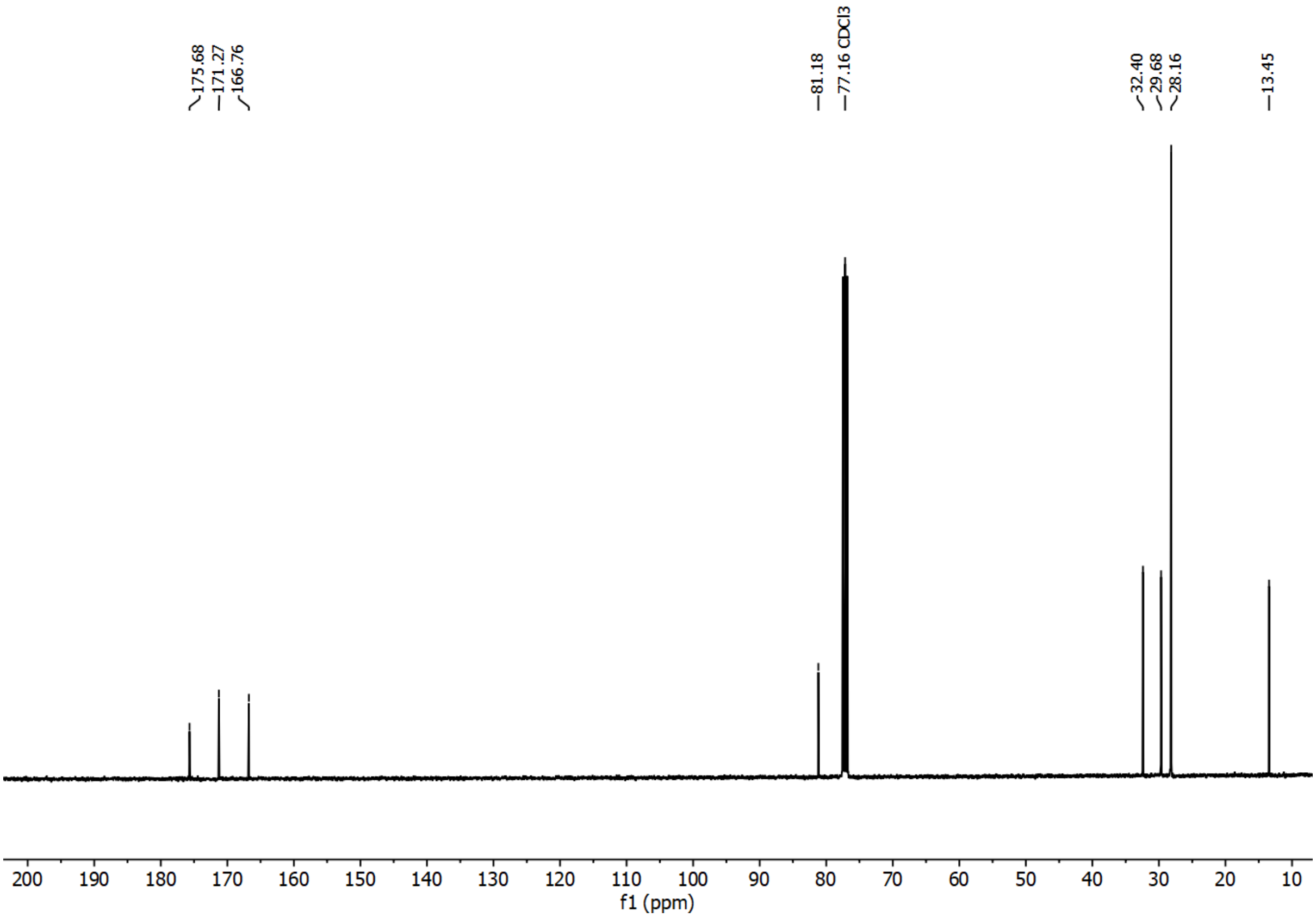

^{1}H-NMR and ^{13}C-NMR of 3-methylthio-6-(3-*tert*-butylcarboxypropyl)-1,2,4,5-tetrazine (**3**).

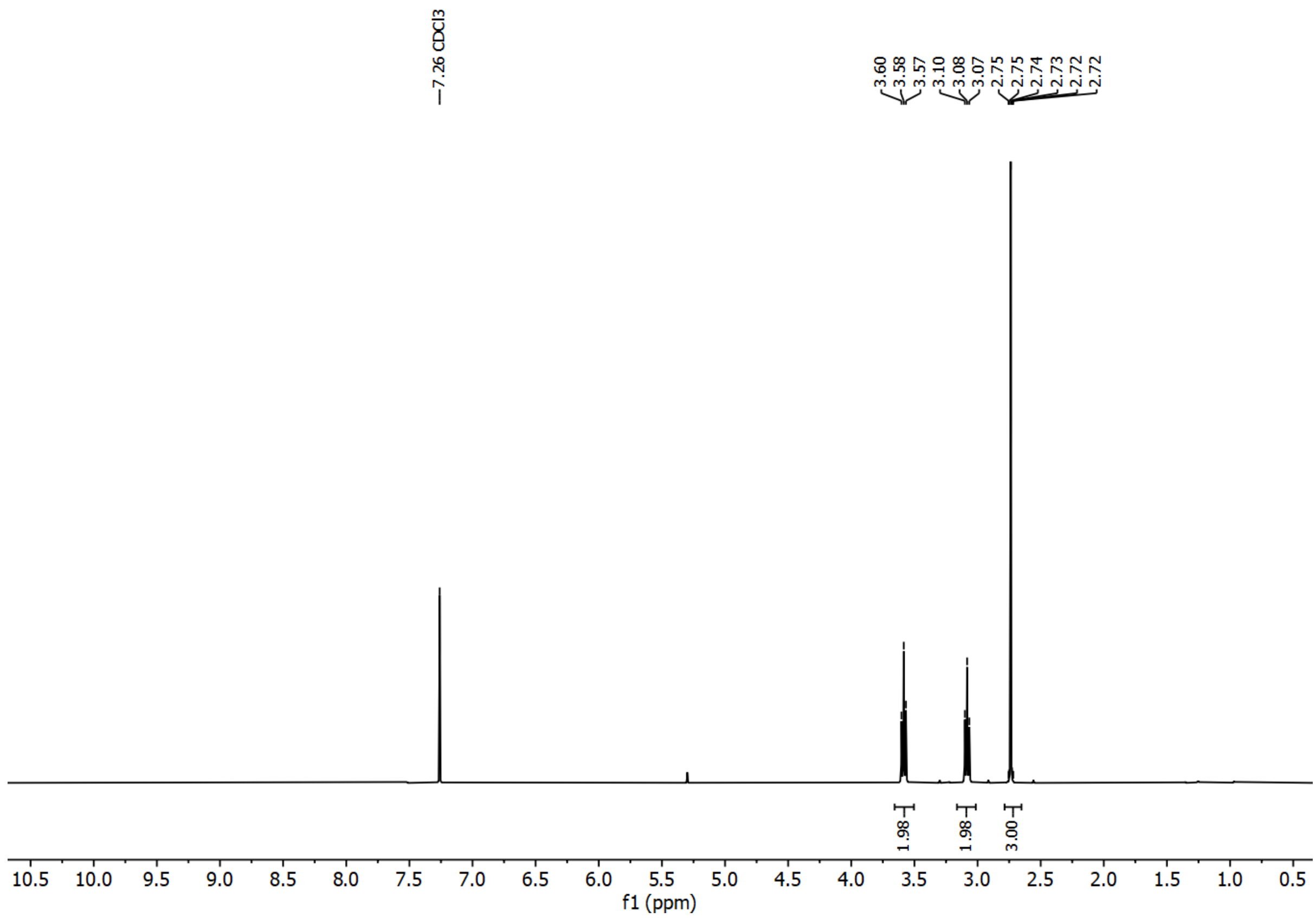

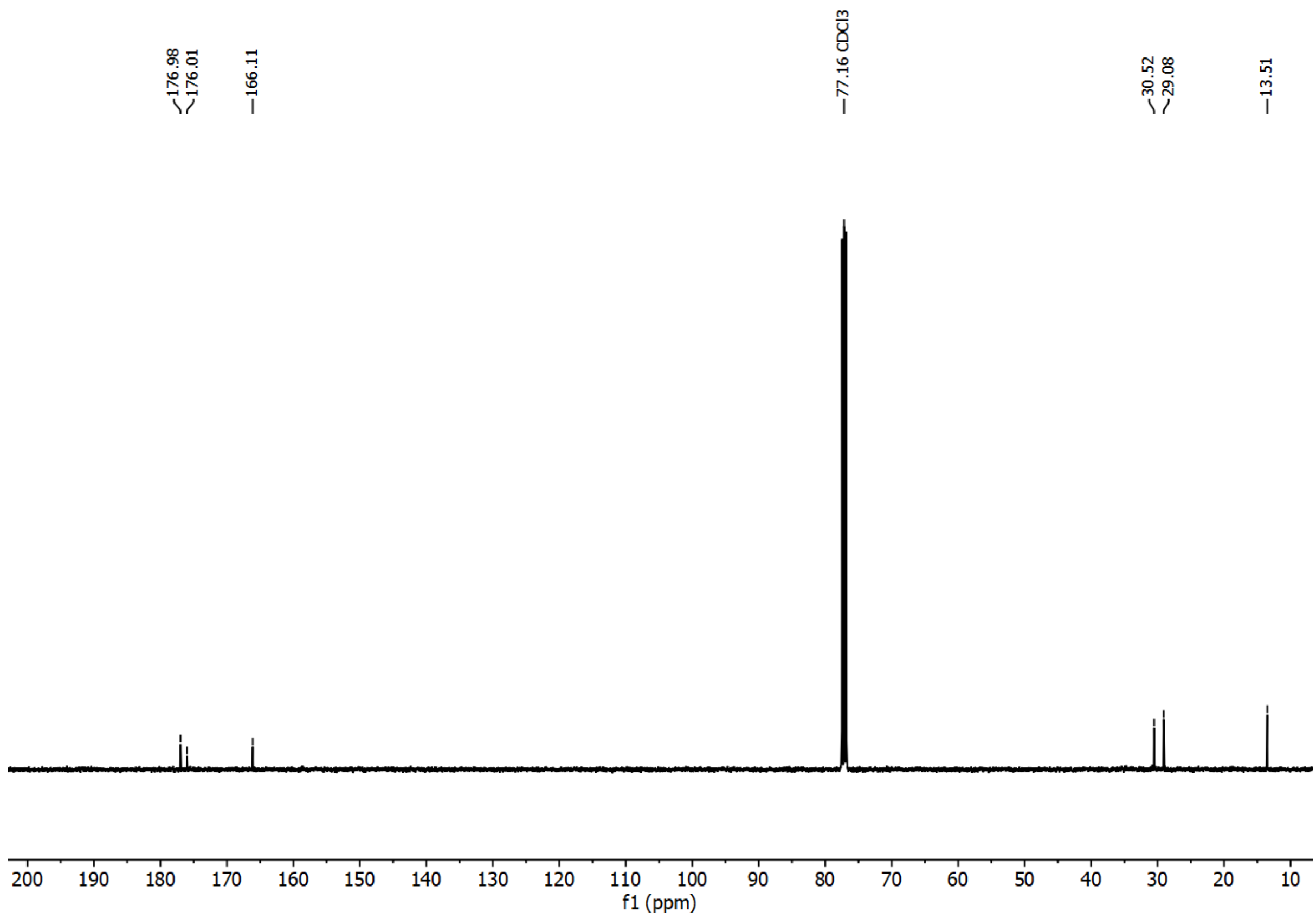

^{1}H-NMR and ^{13}C-NMR of 3-methylthio-6-(3-carboxypropyl)-1,2,4,5-tetrazine (**4**).

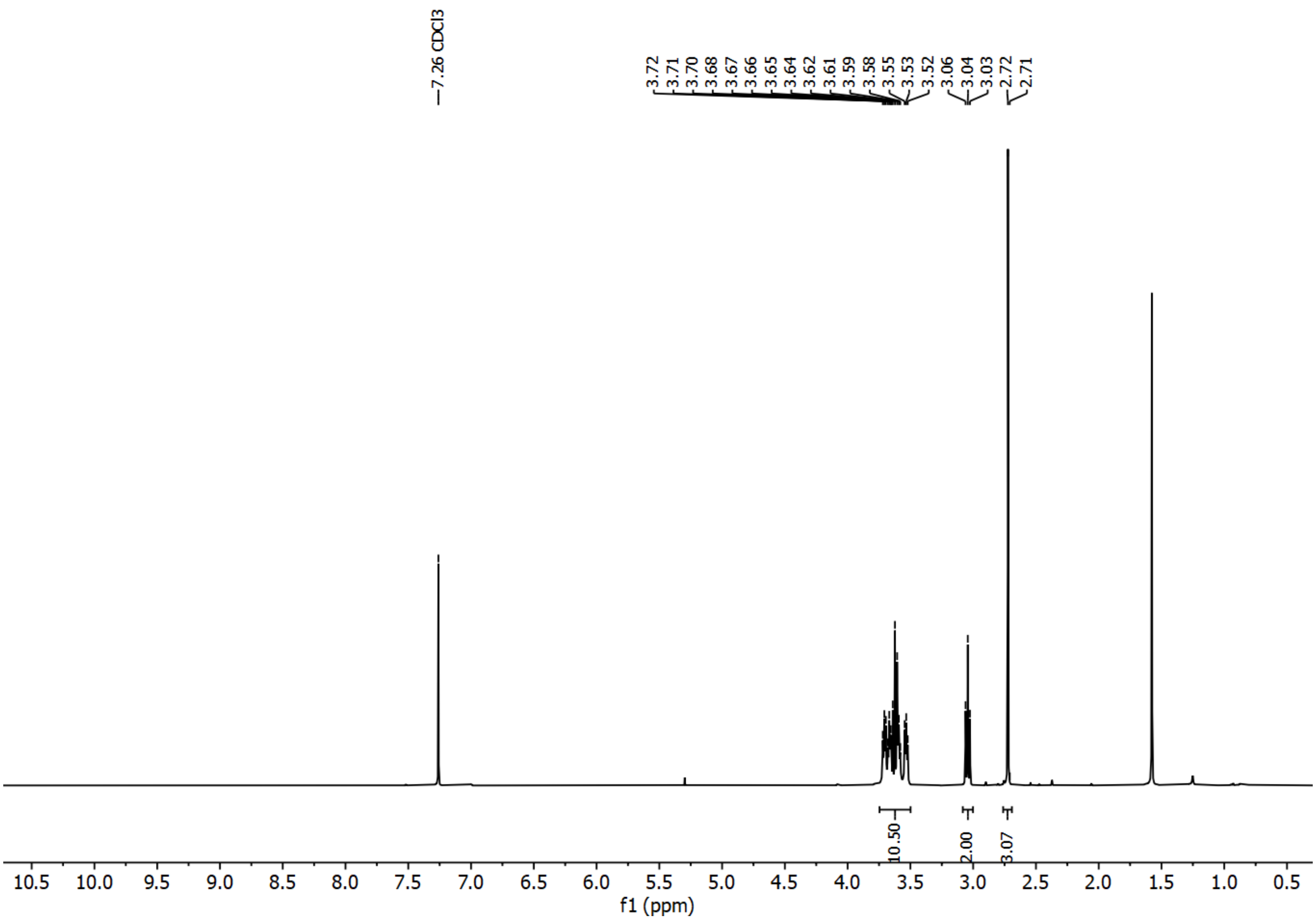

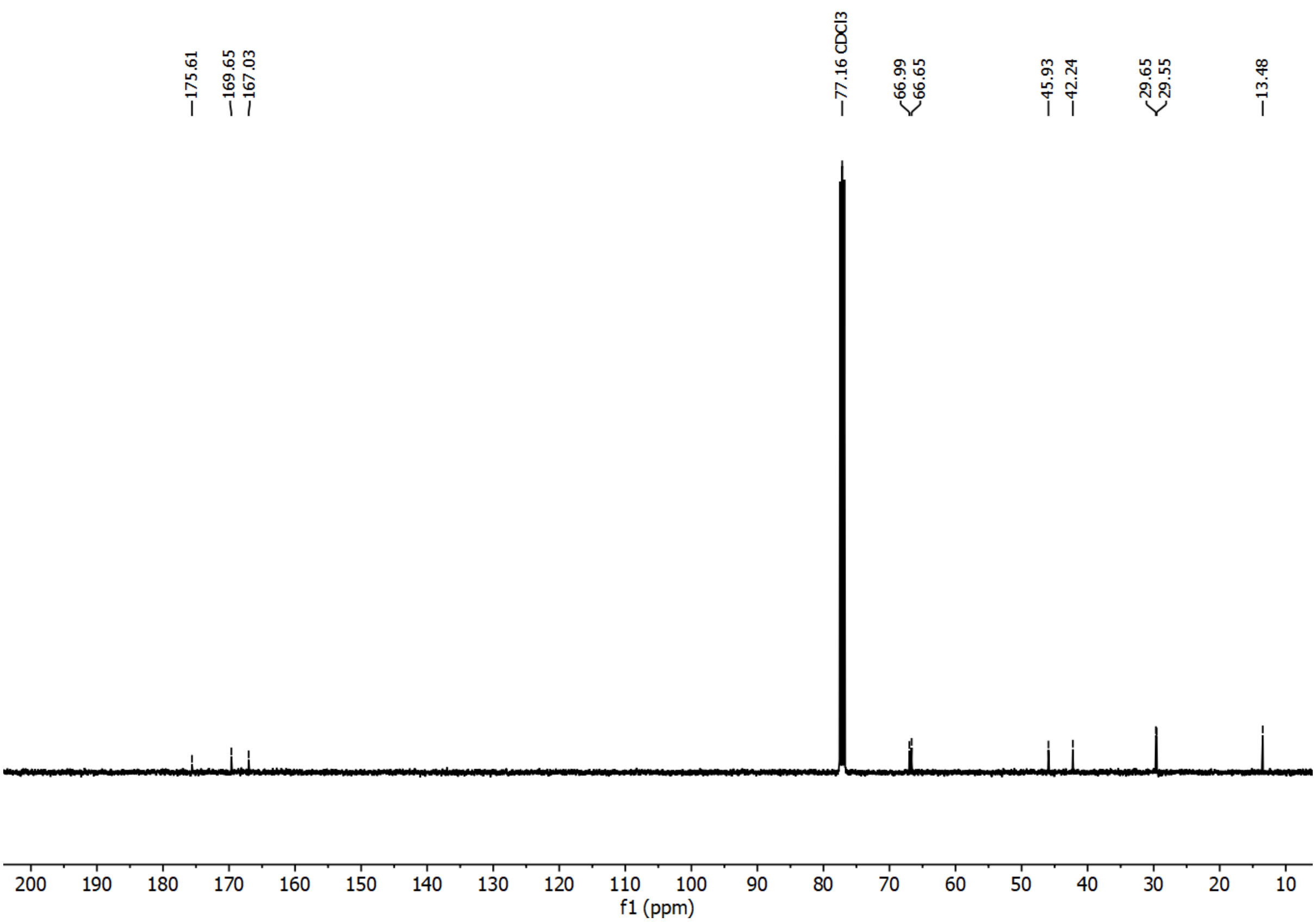

^{1}H-NMR and ^{13}C-NMR of 3-methylthio-6-(3-morpholin-4-ylpropan-2-one)-1,2,4,5-tetrazine (**5**)
